# Integrating Epigenomic Elements and GWASs Identifies *BDNF* Gene Affecting Bone Mineral Density and Osteoporotic Fracture Risk

**DOI:** 10.1038/srep30558

**Published:** 2016-07-28

**Authors:** Yan Guo, Shan-Shan Dong, Xiao-Feng Chen, Ying-Aisha Jing, Man Yang, Han Yan, Hui Shen, Xiang-Ding Chen, Li-Jun Tan, Qing Tian, Hong-Wen Deng, Tie-Lin Yang

**Affiliations:** 1 Key Laboratory of Biomedical Information Engineering of Ministry of Education, School of Life Science and Technology, Xi’an Jiaotong University, Xi’an 710049, P. R. China; 2 School of Public Health and Tropical Medicine, Tulane University New Orleans, LA 70112, USA; 3 Laboratory of Molecular and Statistical Genetics, College of Life Sciences, Hunan Normal University, Changsha 410081, P. R. China

## Abstract

To identify susceptibility genes for osteoporosis, we conducted an integrative analysis that combined epigenomic elements and previous genome-wide association studies (GWASs) data, followed by validation at population and functional levels, which could identify common regulatory elements and predict new susceptibility genes that are biologically meaningful to osteoporosis. By this approach, we found a set of distinct epigenomic elements significantly enriched or depleted in the promoters of osteoporosis-associated genes, including 4 transcription factor binding sites, 27 histone marks, and 21 chromatin states segmentation types. Using these epigenomic marks, we performed reverse prediction analysis to prioritize the discovery of new candidate genes. Functional enrichment analysis of all the prioritized genes revealed several key osteoporosis related pathways, including Wnt signaling. Genes with high priority were further subjected to validation using available GWASs datasets. Three genes were significantly associated with spine bone mineral density, including *BDNF, PDE4D*, and *SATB2*, which all closely related to bone metabolism. The most significant gene *BDNF* was also associated with osteoporotic fractures. RNA interference revealed that *BDNF* knockdown can suppress osteoblast differentiation. Our results demonstrated that epigenomic data could be used to indicate common epigenomic marks to discover additional loci with biological functions for osteoporosis.

Osteoporosis is a major public health problem due to the aging population globally. This common skeletal disease is characterized by low bone mass, poor bone quality, and an increased predisposition to fractures^1^. Osteoporosis is diagnosed clinically through the measurement of bone mineral density (BMD), which is the most widely used predictor of fractures^2^,^3^. Therapeutic decisions that are aimed at preventing fractures are often based on BMD measurement.

BMD is known to be highly heritable, with heritability estimates between 0.6–0.8^4^. Osteoporotic fracture, an endpoint clinical outcome of osteoporosis, also has moderate heritability of 0.5–0.7^5^,^6^. During the past few years, genome-wide association studies (GWASs) have been demonstrated to be an effective strategy for genetic dissection of human complex diseases/traits^7^. Through this strategy, over 100 novel genetic loci have been successfully identified for osteoporosis^8^. However, the genetic variants identified so far together explain only a small proportion of the heritability for osteoporosis^9^. Due to the modest genetic effect size and inadequate statistical power, true association signals may not be discovered with the use of a stringent genome-wide significance threshold alone^10^. It is likely that a sizable proportion of those rejected associations are false negative, and methods of interpretation are needed to recognize such associations.

In addition, since most associated SNPs reported by GWASs reside within intronic or intergenic regions^11^, and the majority of the GWASs have not provided much information beyond statistical signals, it is difficult to elucidate the functional mechanisms for those novel susceptibility loci in determining the phenotype. Strikingly, these GWASs SNPs are usually involved in regulating gene expression via some common regulatory or functional elements^12^,^13^,^14^. Finding such common regulatory elements could offer new insight into the biological link between the susceptibility SNPs and the phenotype, and therefore provide new therapeutic targets in the era of epigenomic drug development^15^,^16^. Fortunately, the Encyclopedia of DNA Elements (ENCODE) project has provided a rich source of regulatory data (i.e. epigenomic elements) for genome annotation^17^, mainly including transcription factor binding sites (TFBSs), chromatin states segmentation, and histone modification marks. TFBSs are the binding sites found in DNA for transcription factors (TFs), which are linked to transcriptional regulation. Chromatin state segmentation is a descriptive classification of chromatin, such as “enhancer” or “repressor”. Histone modification could impact gene expression by altering chromatin structure or recruiting histone modifiers. Gene expression is regulated by the interplay of these epigenomic elements. For instance, TF occupancy is linked to diverse chromatin features characterized by distinct histone composition, histone modifications and by binding of specific proteins. Depending on cellular contexts, a TF can bind to different TF occupancy and regulate different gene expression programs^18^. Recent studies have found that phenotype-associated SNPs are enriched in regions with epigenomic elements implicated in gene regulation^17^,^19^, which reminds us that it is necessary to incorporate epigenomic elements information to interpret GWASs data. Moreover, prioritizing candidate genes with those common epigenomic elements could help predict new susceptibility loci for a particular disease/trait.

Therefore, in this study, we hypothesized that common disease-specific epigenomic elements could identify additional susceptibility genes for disease. Through integrating epigenomic elements data, GWASs data, and phenotypic data, we (1) analyzed the features of epigenomic elements for all the osteoporosis-associated genes implicated by GWASs, including TFBSs, chromatin states segmentation, and histone modification marks defined by ENCODE; (2) found common epigenomic marks associated with osteoporosis and prioritized all genes by these epigenomic marks to predict new susceptibility genes; (3) validated the predicted genes for associations with osteoporosis using available GWAS datasets, including GEFOS (Genetic Factors for Osteoporosis Consortium) dataset, and four GWASs samples from in-house studies. (4) explored the functional roles of the newly identified genes involved in bone cells. Our results demonstrated that epigenomic data could be used to indicate common epigenomic marks to identify susceptibility genes with biological functions for osteoporosis, which would therefore improve the power to detect associations, and offer strategies for developing new therapeutic targets.

## Results

### Osteoporosis-associated genes set

We obtained a total of 259 osteoporosis-associated genes from the GWAS Catalog and PheGenI database (Supplemental Table S1). These genes were supplied to pathway enrichment analysis using the STRING online tool (http://string-db.org/). Twelve significant pathways were found (*P* < 0.05, Supplemental Table S2). Expectedly, these osteoporosis-associated genes tend to enriched in well-known osteoporosis related pathways, such as Wnt signaling, Hedgehog signaling, osteoclast differentiation, and MAPK signaling pathways.

### Identification of epigenomic elements enriched/depleted in osteoporosis-associated genes

**Figure 1:**
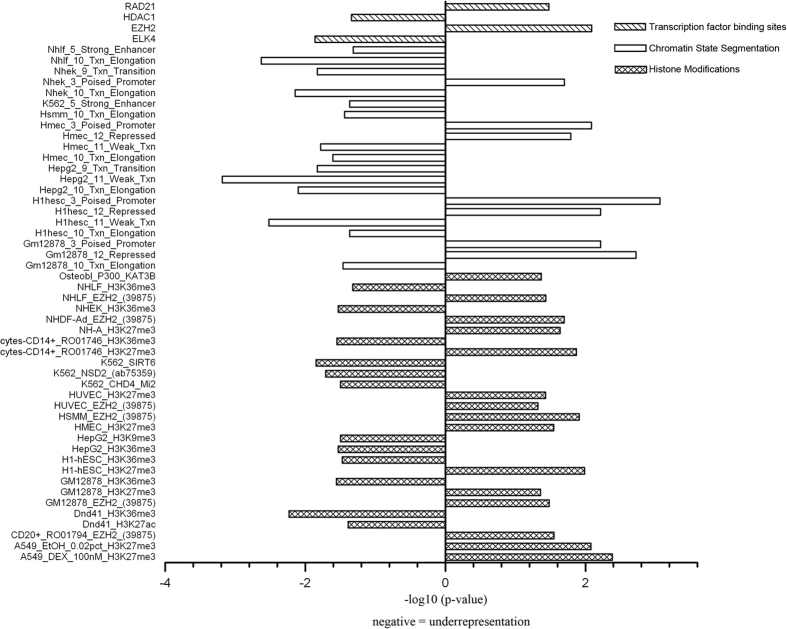
Enrichment/depletion of 52 epigenomic elements in the promoters of osteoporosis-associated genes. The x-axis denotes the −log10 transformed enrichment *P*-values for each element.

We examined whether or not any of the epigenomic elements were enriched or depleted in the osteoporosis-associated genes. Three groups of epigenomic elements were used in the analyses, including 161 TFBSs, 135 chromatin states, and 273 histone marks, which are summarized in more detail in Supplemental Table S3. A total of 52 epigenomic elements were identified to be significantly enriched or depleted in the osteoporosis-associated genes (Fig. 1).

For the TFBSs, we identified 2 TFBSs (EZH2 and RAD21) for enrichment, and 2 TFBSs (ELK4 and HDAC1) for depletion in the promoters of osteoporosis-associated genes, respectively (Fig. 1, Supplemental Table S3).

We then analyzed cell type-specific epigenomic elements which regulate the accessibility of chromatin, such as chromatin states and histone marks. Multiple cell types from ENCODE were used to collect these epigenomic data (Supplemental Table S4). For the chromatin states, 21 out of the 135 chromatin states segmentation types were identified to be enriched or depleted in the promoters of osteoporosis-associated genes (Fig. 1, Supplemental Table S3). Specifically, both “poised promoter” and “repressed” chromatin regions were significantly enriched in 3 cell lines, including Gm12878 (Epstein-Barr Virus transformed B-lymphoblastoid), H1hesc (Human Embryonic Stem Cells), and Hmec (Human Mammary Epithelial Cells). On the other hand, depletions of “transcriptional elongation”, “weak transcribed”, and “transcriptional transition” regions were found in several cell lines, including Gm12878, H1hesc, Hmec, Hepg2 (Hepatocellular Carcinoma), Nhek (Normal Human Epidermal Keratinocytes), Hsmm (Human Skeletal Muscle Myoblasts), and Nhlf (lung fibroblasts). “Strong enhancer” region was depleted in Nhlf and K562 chronic myelogenous leukemia cell lines.

Analysis of histone modification marks identified 27 histone marks enriched or depleted in the promoters of osteoporosis-associated genes (Fig. 1, Supplemental Table S3). Specifically, “EZH2” mark was enriched in 6 cell lines, including Hsmm, B cells, Nhdf-Ad (Normal Human Adult Dermal Fibroblasts), Gm12878, Nhlf, and Huvec (Human Umbilical Vein Endothelial Cells). “KAT3B” mark was enriched in osteoblast. A repressive H3K27me3 mark^20^ was enriched in 7 cell lines, including GM12878, A549 (epithelial cell line from a lung carcinoma tissue), Monocytes, H1hesc, NH-A (astrocytes), Hmec, and Huvec, while an activating H3K36me3 mark was depleted in 7 cell lines, including Dnd41 (T cell), Nhek, monocyte, GM12878, Hepg2, H1hesc, and Nhlf. The enrichment of H3K27me3 and depletion of H3K36me3 were mostly non-cell type-specific.

### Reverse prediction suggests new susceptibility genes for osteoporosis

Given that the identified epigenomic elements enriched/depleted in osteoporosis-associated genes could reflect osteoporosis-relevant regulatory factors, we performed reverse prediction analysis to prioritize all genes by these epigenomic features, which would suggest additional and/or novel susceptibility genes with high tendency to influence osteoporosis. We calculated a total score for each gene to make a ranking list. The top 20 genes ranked by the total scores are presented in Table 1.

### Functional annotation and pathway analysis

To explore whether genes identified by the reverse epigenomic analysis are relevant to osteoporosis and may provide novel targets to osteoporosis, we conducted GSEA on all genes prioritized by the total scores. The results were similar as the original osteoporosis-associated genes. As shown in Table 2, we identified 36 significant pathways (FDR *P* < 0.05). The enriched pathways related to osteoporosis are particularly interesting, including Wnt signaling, calcium signaling, Hedgehog signaling, MAPK signaling, and TGF-β signaling pathways.

**Figure 2:**
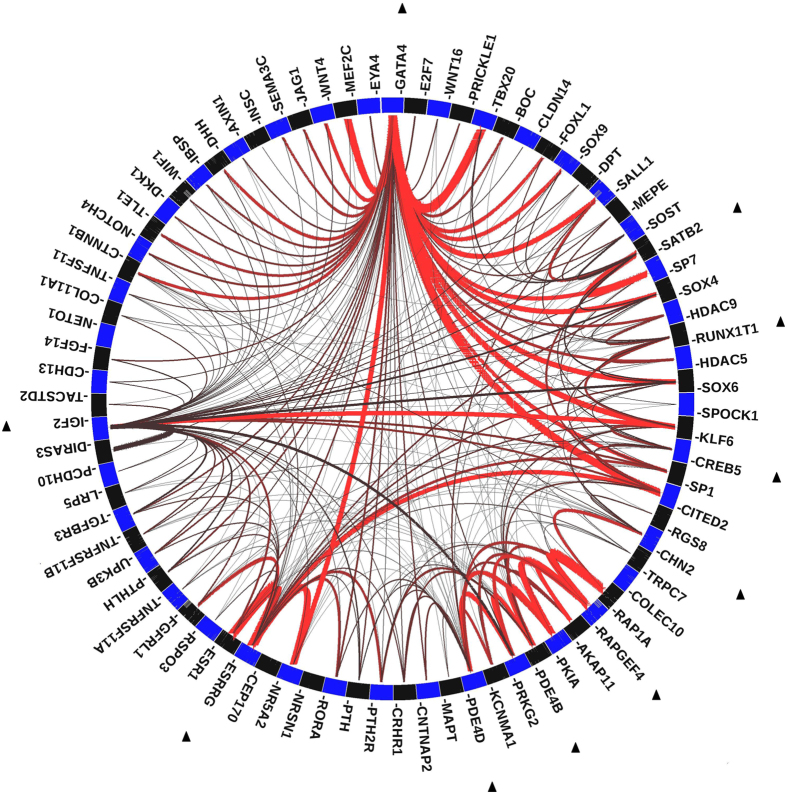
Connections between the top ranking predicted genes and the known osteoporosis-associated genes. Ten out of the 20 predicted genes, which are marked by black triangle, are connected with 69 known osteoporosis-associated genes. The lines between genes represent individually significant connections that contribute to the positive signal, with the thickness of the lines being inversely proportional to the probability that a literature-based connection would be seen by chance.

We further applied GRAIL analysis to investigate potential connections between the top 20 genes from reverse prediction analysis and the 259 known osteoporosis-associated genes. As shown in Fig. 2, 10 of the 20 predicted genes were connected with 69 known osteoporosis-associated genes.

### Validation at the population level

To confirm the relationship between the predicted genes with osteoporosis, we examined associations between the top 20 genes and BMD in four available GWAS BMD datasets, of which one was from the GEFOS dataset and the other three were from our own group (KCOS, OOS and COS). The basic characteristics of our samples are summarized in Table 3. Among the top 20 genes, 3 genes, including brain-derived neurotrophic factor (*BDNF*), phosphodiesterase 4D (*PDE4D*), and SATB homeobox 2 (*SATB2*), were successfully validated for associations with spine BMD. The detailed association results are summarized in Table 4. According to meta-analysis over the 4 GWAS datasets, two SNPs of *BDNF*, rs7124442 (3′UTR variant) and rs11030119 (intron variant), achieved *P* values of 2.47 × 10^−5^ and 7.65 × 10^−5^, respectively. Two SNPs of *PDE4D*, rs2938780 and rs2963826 (both intron variants), achieved *P* values of 1.15 × 10^−4^ and 1.27 × 10^−4^, respectively. And two SNPs of *SATB2*, rs895526 and rs6704641 (both intron variants), achieved *P* values of 1.25 × 10^−4^ and 1.28 × 10^−4^, respectively. After multiple testing corrections, all of these 6 SNPs from 3 genes remained significant (adjusted *P* < 0.05), and the directions of their effects on spine BMD were totally consistent across different studies. The heterogeneity test between studies showed that there was no heterogeneity for all of the SNPs we identified (all *Q*_het_ *P *> 0.05, *I*^*2*^ = 0) (Table 4).

For the above 6 significant SNPs, we also tested for associations with osteoporotic fractures in the CFS sample (Table 5). Both SNPs of *BDNF* were found to be significantly associated with osteoporotic fractures (rs11030119: *P *= 0.024; and rs7124442: *P *= 0.042). These two SNPs were in high LD with each other (pairwise LD *r*^2^* *= 0.9). The minor alleles of both SNPs have protective effects on fractures, with the odds ratio (OR) estimated to be 0.58 (95% confidence interval (CI): 0.35–0.96) for rs11030119, and 0.67 (95% CI: 0.45–1.00) for rs7124442, respectively. The effects on fractures risk for both SNPs were totally consistent with their associations with increased BMD values.

To explore the functional relevance of the identified 6 significant SNPs, we performed cis-eQTLs analysis in 462 unrelated human LCLs samples from 1000 Genome Project. Although all of these 6 SNPs were not associated with expression levels of their transcript, we found several surrogate SNPs, which were in high LD with the two significant SNPs (rs7124442 and rs11030119) in *BDNF*, significantly associated with *BDNF* mRNA expression levels (Table 6).

### Functional Assays

**Figure 3:**
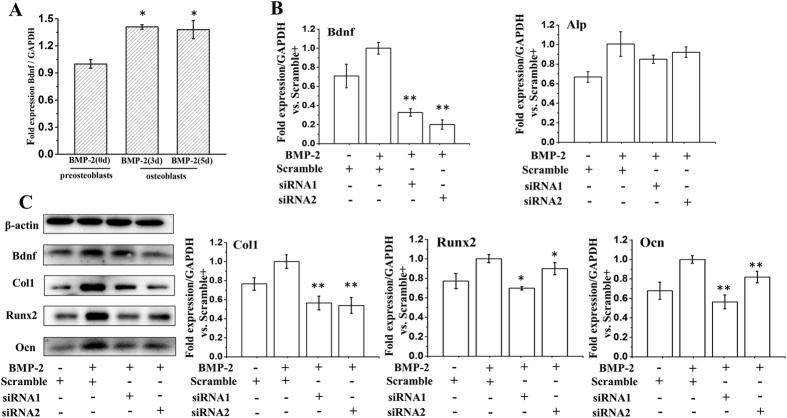
Functional analysis of siRNA mediated *BDNF* knockdown in osteoblasts. (**A**) Cells were grown with 200 ng/ml rhBMP-2 for the indicated times, and real-time PCR was performed. *p < 0.05 vs preosteoblasts. (**B,C**) Cells were first transfected with *BDNF* siRNA or scramble siRNA. And then incubating cells with 200 ng/ml rhBMP-2 for additional 2 days, real-time PCR for BDNF, ALP, COL1, RUNX2, OCN (**B**) and western analysis for BDNF, COL1, RUNX2, OCN (**C**) were performed. *p < 0.05 vs the scramble siRNA-treated group in differentiated osteoblast cells. All data are expressed as the mean±SD from three independent experiments.

Based on the significant effect we identified for *BDNF*, we further investigated the function of *BDNF* in bone. *BDNF* is reported to be involved in chondrocyte differentiation, cartilage development and osteogenesis^21^,^22^,^23^. Therefore, we tested the role of *BDNF* in osteoblast biology to assess the function of the identified loci. Real-time PCR revealed that differentiated osteoblasts had higher *BDNF* expression level than pre-osteoblasts (*p* < 0.05) (Fig. 3A). After treatment with siRNA against *BDNF*, we examined the mRNA expression levels of osteoblast differentiation markers, including alkaline phosphatase (ALP), osteocalcin (OCN), collagen type-I (COL1), and runt-related transcription factor 2 (RUNX2)^24^. As shown in Fig. 3B, knockdown of *BDNF* significantly suppressed the expression of marker genes COL1, RUNX2, and OCN, compared with the control siRNA treated group in differentiated osteoblast cells (*p* < 0.05). Western blot analysis gave similar results (Fig. 3C), suggesting that *BDNF* may stimulate osteoblast differentiation, resulting in increased bone formation.

## Discussion

With GWAS becoming a convenient and powerful tool for genetic decipherment of complex diseases, an arising new challenge is how to utilize the genomic data efficiently to interpret the GWASs results and better understand the disease mechanisms. The ENCODE project^17^ has provided a wealth of various functional elements data, which can be used to the design, analysis and interpretation of GWASs, and therefore improve our knowledge of human diseases processes^25^. In this study, through integrating epigenomic elements and GWASs data, we identified a set of distinct epigenomic elements associated with osteoporosis. The further reverse analysis based on these epigenomic features predicted a ranking list of candidate genes for osteoporosis, and we successfully identified *BDNF* as a susceptibility gene for BMD and osteoporotic fractures, which highlights the efficiency of finding missing heritability of osteoporosis by reasonably prioritizing genes using epigenomic data.

For the epigenomic elements analysis, we didn’t restrict our analysis to any given cell types. It is because that the osteoporosis-associated genes identified by GWASs are disease-specific, not cell type-specific. Moreover, osteoporosis is a systematic metabolic disease, which could be caused by a number of diseases, including diabetes, hyperthyroidism, gastrointestinal disorders, kidney disease, rheumatoid arthritis, and systemic lupus erythematosus. It might lose some information by focusing on any given cell types. For the identified significant epigenomic marks, EZH2 (enhancer of zeste homolog 2) has been reported as a transcription repressor through H3K27me3^26^. Previous studies have revealed that EZH2 could interact with Wnt signaling^27^,^28^,^29^, which is a crucial pathway to bone biology and development. Interestingly, we found that most of the enriched epigenomic elements for osteoporosis are repressed or inactive marks, such as “poised promoter”, “repressed” chromatin regions, EZH2, and repressive H3K27me3 mark, which suggests that osteoporosis-associated genes tend to be affected by repressive or inactive epigenomic marks, and disruption of these transcriptionally inactive or repressive state might be a factor in the disease.

We suggest a list of novel candidate genes for osteoporosis. Among which, three genes (*BDNF, PDE4D*, and *SATB2*) were confirmed for association with spine BMD in four GWAS datasets. Especially, *BDNF* was also associated with osteoporotic fractures. *BDNF* encodes a member of the neurotrophin family of growth factors, which are related to the canonical nerve growth factor. BDNF is required for differentiation and survival of specific neuronal subpopulations in both central and peripheral nervous systems^30^. Moreover, BDNF mRNA was previously reported to be expressed in murine osteoblasts (MC3T3-E1 cells)^31^. Several studies have shown that BDNF plays a role in chondrocyte differentiation, cartilage development and osteogenesis^21^,^22^,^23^. A phosphorylation-related SNP rs6265 in *BDNF* has been identified to be associated with BMD in humans^32^. We explored the role of *BDNF* in osteoblast biology and found that siRNA mediated *BDNF* knockdown can suppress the expression of osteoblast differentiation markers, suggesting that *BDNF* may stimulate osteoblast differentiation, resulting in increased bone formation. Pathway analysis revealed that *BDNF* is involved in the MAPK signaling pathway, which plays an essential role in osteoblast differentiation and skeletal development^33^,^34^,^35^. Modulation of bone formation by *BDNF* may influence fracture risk by affecting both bone mass and bone quality. The other two genes also have potential connection with bone or osteoporosis. *PDE4D* encodes cyclic AMP-dependent phosphodiesterase 4D. PDE4D selective inhibitors can promote osteoblast differentiation in progenitor cells^36^ and increase bone mass by promoting bone formation in normal mice^37^. A previous genetic association study identified a variant in *PDE4D* associated with lumbar spine BMD in females^38^. *SATB2* encodes a nuclear matrix-associated transcription factor and epigenetic regulator that plays a critical role in osteoblast lineage commitment^39^. Targeted knockout of *SATB2* in mice could result in impaired osteoblast differentiation and craniofacial skeletal defects^40^. Recent studies suggested SATB2 as a novel sensitive marker of osteoblastic differentiation^41^,^42^. Together, taking into account of the above lines of biological evidence, our findings further highlights the importance of these genes to the pathogenesis of osteoporosis, and also support our hypotheses that epigenomic data could be used to predict susceptibility genes with functional information for diseases.

Our study has several implications compared with conventional GWAS strategies. First, candidate gene prioritization strategies by epigenomic data could increase the prior probability of an association test, and therefore increase the power of detecting bona fide associations in a study of a given size, which may discover genes that would be missed by traditional association studies relying on strictly *P* value driven approaches. Second, incorporating epigenomic regulatory information may provide more insight into disease biology and offer strategies for therapeutic development. The susceptibility genes we identified have close relationship with bone metabolism. Functional enrichment analysis of the genes prioritized by the epigenomic elements revealed osteoporosis related pathways, including Wnt signaling, Hedgehog signaling, and MAPK signaling pathways, which are consistent with our prior understanding of the pathophysiology of osteoporosis, and meanwhile implicate the feasibility of our study. Third, we confirmed the significant associations of 3 predicted genes in our 3 GWAS datasets and GEFOS dataset. Specifically, GEFOS is the largest dataset for osteoporosis GWAS meta-analysis^9^. Thus, our association results are robust and may provide convergent validity for our findings.

Our current study also has some limitations. For example, in the gene validation stage, we arbitrarily selected the top 20 genes and further remained significant after multiple testing corrections in four GWAS datasets. Thus, it is likely that some genes that contribute to osteoporosis susceptibility but did not meet our selection criteria could have been missed. Second, for the epigenomic elements analyses, we focused on the promoter regions of genes, since promoter is a critical regulatory region that can work in concert with many other regulatory elements to direct the level of transcription of a given gene. It is easy to find regulatory commonalities, but might neglect some potential meaningful epigenomic elements located on other regions of genome.

In conclusion, through the integrated analysis of GWASs and epigenomic data, we identified a set of significant regulatory elements enriched in osteoporosis-associated genes. We also discovered *BDNF* as a susceptibility gene implicated in osteoporosis that is biologically meaningful. Beyond generating a list of associated SNPs by statistical signals, our findings demonstrate that an integrative approach combining GWASs and epigenomic profiling could lead to the identification of additional loci with functional information underlying osteoporosis. The genes may provide future targets for research into the etiology and treatment of osteoporosis.

## Materials and Methods

### Acquisition of osteoporosis-associated genes

We used the National Human Genome Research Institute (NHGRI) GWAS Catalog^43^ (www.genome.gov/gwastudies downloaded on Apr 20, 2015) and Phenotype-Genotype Integrator (PheGenI) database^44^ (http://www.ncbi.nlm.nih.gov/gap/phegeni) to obtain a list of osteoporosis-associated genes, using osteoporosis related phenotypes including BMD, fractures, femoral neck bone geometry, hip bone size, and spine bone size.

### Functional annotation

We used ENCODE data drawn from the UCSC genome browser to conduct functional annotation for the genomic regions of interest^45^. In this study, we focused on promoter regions of osteoporosis-associated genes. An in-house Perl script was used to extract the promoter regions, which were defined as 2,000 nucleotides upstream of a gene’s transcription start site. For the genes with more than one transcript, the pipeline extracted the promoters for each transcript, and merged overlaps into a single promoter. The annotated genomic features can be classified into three groups of epigenomic elements, including TFs obtained experimentally by ChIP-seq, histone modifications by ChIP-seq, and chromatin state segmentation by hidden Markov model (HMM) from ENCODE. A total of 569 epigenomic elements were used in the analysis. The data from multiple cell lines were used.

### Enrichment analysis of epigenomic elements

We first calculated the total number of promoters of osteoporosis-associated genes annotated by the 569 epigenomic elements obtained above. The annotation was defined that if the promoter overlaps with each epigenomic element for at least 1 nucleotide, it means that the promoter is annotated by this element^46^. If a given promoter overlaps with the same epigenomic element for more than 1 time, it is only counted once to reflect the fact of overlap. Then, using the promoters of all genes on genome as a background, we randomly selected the same number of promoters as those in the osteoporosis-associated genes set to perform random sampling, which could distinguish regulation of one set of genes from another. Such random sampling was repeated 1,000 times to estimate the average number and variance of random annotation. Compared with the random sampling results, we implemented Fisher’s exact test to identify epigenomic elements that was significantly over-represented or under-represented in the osteoporosis-associated genes. For easier comparison and visualization, *P* values were transformed into logarithm (log_10_[*P*] for under-represented; −log_10_[*P*] for over-represented). As a control group, we also randomly selected a genes set of the same size as the osteoporosis-associated genes to conduct the above process.

### Reverse prediction

To predict new candidate genes for osteoporosis, we analyzed the promoters of other genes to evaluate whether they shared similar set of epigenomic elements as those promoters of osteoporosis-associated genes. The promoters of all genes were annotated for the presence of the significant epigenomic elements obtained by the above enrichment analysis. For each gene, we first counted the times of its promoter annotated by each of the significant epigenomic elements. Then we weighted the counts of each element by the corresponding transformed *P* values. By summing up the weighted counts on each promoter, we acquired the total score denoting each gene to prioritize the importance of genes.

### Functional annotation and pathway analysis

We ranked all genes based on the scores obtained from the reverse epigenomic analysis. The ranked gene list was supplied to gene set enrichment analysis (GSEA)^47^ pre-ranked analysis with default parameters. c2 KEGG (curated gene sets from KEGG pathway databases) were used for the analysis. We used the Gene Relationships Across Implicated Loci (GRAIL) text-mining algorithm^48^ to investigate connections between the predicted new genes and the known osteoporosis-associated genes identified by GWASs.

### Validation in GWAS datasets

To validate the predicted candidate genes at the population level, we took advantage of the available five GWAS datasets, of which one was from the published GEFOS dataset and the other four were from our own group. All the studies related to the datasets were approved by the respective institutional ethics review boards and all participants provided signed informed-consent documents. The related information is described in detail as follows.

### GWAS datasets

#### GEFOS dataset

We checked the SNPs of the interested genes for their association signals with femoral neck and lumbar spine BMD in the GEFOS (Genetic Factors for Osteoporosis Consortium) dataset (http://www.gefos.org). GEFOS is the largest GWAS meta-analysis to date in the bone field, including 17 GWASs and 32,961 individuals of European and East Asian ancestry^9^.

#### Four in-house GWAS samples

Our own GWAS datasets include three BMD samples and one fracture sample: 1) Kansas City Osteoporosis Study (KCOS) with 2,286 unrelated individuals of European ancestry; 2) Omaha osteoporosis study (OOS) with 987 unrelated individuals of European ancestry; 3) Chinese Osteoporosis Study (COS) with 1,627 unrelated Chinese of Han ethnicity; and 4) Chinese Fractures Study (CFS) with 350 cases with osteoporotic hip fractures and 350 elderly healthy controls. The description of each study has been detailed in our previous studies^49^,^50^,^51^.

### Phenotype measurements

For our own three GWAS BMD samples, BMD (g/cm^2^) at spine and femoral neck (FN) was measured with dual energy x-ray absorptiometry (DXA) using Hologic 4500 W machines (Hologic Inc., Bedford, MA, USA) that were calibrated daily. For the GEFOS samples, BMD was measured with DXA scanners using Hologic (Hologic Inc., Bedford, MA, USA) or Lunar scanners (Lunar Corp., Madison, WI, USA). Raw BMD values were adjusted by significant covariates including sex, age, weight and height. To adjust for potential population stratification, the first ten principal components emerging from the EIGENSTRAT analyses were also included as covariates^52^.

### Genotyping and quality control

Samples from KCOS and COS were genotyped using Genome-Wide Human SNP Array 6.0 (Affymetrix Inc., Santa Clara, CA, USA), and sample from OOS and CFS were genotyped using the Affymetrix Human Mapping 500 K array set, according to the manufacturer’s protocols. The details of genotyping for each sample have been described in our previous studies^49^,^50^,^51^. Quality control of genotype data were implemented with PLINK^53^, with the following criteria applied: individual missingness < 5%, SNP call rate >95%, and Hardy-Weinberg equilibrium (HWE) *P*-value < 0.0001.

### Association analyses

For the KCOS and COS samples, a linear regression implemented in PLINK^53^ was fitted to test for association assuming an additive inheritance model. For the OOS and CFS samples, IMPUTE program^54^ was utilized to impute the genotypes of SNPs detected on Array 6.0 but not on 500 K array set based on HapMap data (release 22). To ensure the reliability of imputation, all imputed SNPs reached a calling threshold of 0.90, i.e., a 90% probability that an imputed genotype is true. SNPTEST^54^ was used to examine associations in these samples. Summary statistics of associations from each GWAS BMD sample were combined to conduct meta-analysis using the METAL software package^55^, taking into account the square-root of each sample size. The between-study heterogeneity was assessed using both the Cochran’s *Q* statistic and the *I*^*2*^ metric. The Benjamini and Hochberg (BH) procedure was used for multiple-testing adjustment.

### Expression quantitative trait locus (eQTL) analysis

We conducted eQTL analysis to evaluate whether the predicted significant SNPs for each locus also influence expression level of their nearest transcript. Gene expression information was obtained from human lymphoblastoid cell lines (LCLs) of 462 unrelated individuals from 1000 Genomes Project^56^. The linear regression model implemented in PLINK^53^ was used to determine associations between expression levels and SNPs. We also included surrogate SNPs with linkage disequilibrium (LD) *r*^2^ > 0.7 with target SNPs in the test.

### Functional Assays

#### Culture of osteoblast cell line

Murine pre-osteoblast MC3T3-E1 cells (ATCC, VA, USA) were cultured in α-minimum essential medium (α-MEM, Invitrogen, CA, USA) containing 10% fetal bovine serum (FBS) and 1% penicillin-streptomycin at 37 °C. To induce osteoblastic differentiation, cells were plated at a density of 5 × 10^4 ^cells/cm^2^ and cultured with 200 ng/mL human recombinant bone morphogenetic protein 2 (rhBMP2, Peprotech, USA). The medium was replaced every other day unless otherwise indicated.

### Transfection with small interfering RNA (siRNA)

When cells confluence reached at 30%, cells were transfected with siRNA against murine brain-derived neurotrophic factor (BDNF) or with non-silencing siRNA (Shanghai GenePharma Co.,Ltd) using X-treme GENE siRNA Transfection Reagent (Roche, NJ, USA) according to the manufacturer’s instruction. Briefly, after incubating MC3T3-E1 cells with siRNA-reagent mixtures in α-MEM containing for 6 hours, 200 ng/ml rhBMP2 were added and cells were incubated for an additional 48 hours. The sequences of siRNA were described in Supplemental Table S5. The efficiency of knockdown was at least 70% as confirmed by analyses of mRNA levels.

### Semi-quantitative RT-PCR and Real-time PCR

Total RNA was isolated using the TRIzol reagent (Invitrogen, CA, USA), and complementary DNA (cDNA) was synthesized using the Super Scripts II First-Strand cDNA synthesis kit (Invitrogen, CA, USA) according to the manufacturer’s instructions. Semi-quantitative RT-PCR experiments were optimized for the number of cycles to ensure that the amplifications were analyzed within an exponential phase. We analyzed the expression levels of BDNF, as well as osteoblast differentiation markers, including ALP, OCN, COL1, and RUNX2^24^, by real-time PCR in an Eppendorf Real-time PCR System, using the comparative threshold cycle (Ct) method for relative quantification. The glyceraldehyde-3-phosphate dehydrogenase (GAPDH) gene was used as an endogenous control. The specific primers for indicated genes are presented in Supplemental Table S6.

### Western blot

Total protein was extracted using RIPA buffer (Beyotime Biotechnology, China). Samples were separated by 14% SDS-PAGE, and then transferred onto PVDF membranes (Roche, Germany). After blocking in TBST (Tris buffered saline with 0.1% Tween-20) and 5% non-fat milk and incubated with primary antibodies for BDNF, ALP, OCN, COL1, Runx2, or β-actin (Cell Signaling Technology Inc., USA). Then the membrane was incubated with horseradish peroxidase (HRP)-conjugated goat anti-rabbit secondary antibody (Abcam, MA, USA). Immunoreactivity was detected by enhanced chemiluminescence reaction using Luminata^TM^ Western HRP substrate (Millipore, USA). ECL images were acquired and analysed with the Chemiluminescence Imaging System (CLINX, Shanghai, China).

### Statistical Analysis

Each experiment was repeated independently at least three times, unless indicated otherwise. The results were expressed as the mean ± standard deviation of triplicate independent samples. Student’s t-test was used to examine the differences between the two groups of data. Differences with *p* < 0.05 were considered as statistically significant.

## Additional Information

**How to cite this article**: Guo, Y. *et al*. Integrating Epigenomic Elements and GWASs Identifies *BDNF* Gene Affecting Bone Mineral Density and Osteoporotic Fracture Risk. *Sci. Rep.* **6**, 30558; doi: 10.1038/srep30558 (2016).

## Supplementary Material

Supplementary Information

## Figures and Tables

**Table 1:** Top 20 genes ranked by the total scores from reverse prediction analysis.

Gene	Description	Total score
*PDE4D*	Phosphodiesterase 4D, cAMP-specific	136.16
*ESRRG*	Estrogen-related receptor gamma	135.75
*LINC00461*	Long intergenic non-protein coding RNA 461	117.63
*CHN2*	Chimerin 2	114.19
*IGF2*	Insulin-like growth factor 2	104.99
*LINGO1*	Leucine rich repeat and Ig domain containing 1	100.34
*SATB2*	SATB homeobox 2	99.62
*RUNX1T1*	Runt-related transcription factor 1; translocated to, 1	99.29
*GATA4*	GATA binding protein 4	98.06
*PHACTR3*	Phosphatase and actin regulator 3	97.74
*CREB5*	cAMP responsive element binding protein 5	95.39
*EIF4E3*	Eukaryotic translation initiation factor 4E family member 3	92.64
*PDE4B*	Phosphodiesterase 4B, cAMP-specific	87.49
*SYBU*	Syntabulin (syntaxin-interacting)	87.16
*KCNK10*	Potassium channel, two pore domain subfamily K, member 10	86.73
*BDNF*	Brain-derived neurotrophic factor	86.08
*TRIM36*	Tripartite motif containing 36	85.64
*RTN1*	Reticulon 1	85.13
*RAPGEF4*	Rap guanine nucleotide exchange factor (GEF) 4	85.05
*EGFLAM*	EGF-like, fibronectin type III and laminin G domains	84.80

**Table 2:** Significant pathways by preranked gene set enrichment analysis on all genes prioritized by the predicted total scores.

Name	Size	ES	NES	FDR *P*-value
Neuroactive ligand receptor interaction	270	0.67	2.87	<1.0E-05
Calcium signaling pathway	176	0.58	2.40	<1.0E-05
Hedgehog signaling pathway	56	0.66	2.32	<1.0E-05
Cytokine cytokine receptor interaction	260	0.54	2.29	<1.0E-05
Cell adhesion molecules cams	131	0.58	2.28	<1.0E-05
Basal cell carcinoma	55	0.66	2.27	<1.0E-05
Axon guidance	128	0.56	2.23	<1.0E-05
Autoimmune thyroid disease	50	0.62	2.14	9.15E-05
Melanogenesis	101	0.55	2.13	8.14E-05
Maturity onset diabetes of the young	25	0.71	2.13	7.32E-05
Olfactory transduction	389	0.48	2.11	1.40E-04
Arrhythmogenic right ventricular cardiomyopathy	74	0.57	2.07	2.55E-04
Ecm receptor interaction	84	0.54	1.97	7.09E-04
Gap junction	89	0.52	1.96	7.73E-04
Dilated cardiomyopathy	90	0.51	1.93	1.03E-03
Proximal tubule bicarbonate reclamation	23	0.65	1.93	1.06E-03
Long term depression	70	0.52	1.92	1.10E-03
Hypertrophic cardiomyopathy hcm	83	0.51	1.90	1.42E-03
Melanoma	71	0.52	1.89	1.47E-03
Allograft rejection	35	0.58	1.86	1.84E-03
Type I diabetes mellitus	41	0.57	1.86	1.86E-03
Vascular smooth muscle contraction	115	0.48	1.85	2.07E-03
Wnt signaling pathway	149	0.45	1.83	2.58E-03
Hematopoietic cell lineage	85	0.49	1.83	2.60E-03
Mapk signaling pathway	266	0.43	1.82	2.59E-03
Intestinal immune network for iga production	46	0.54	1.82	2.70E-03
Pathways in cancer	324	0.42	1.80	3.61E-03
TGF beta signaling pathway	85	0.48	1.79	4.06E-03
Graft versus host disease	37	0.54	1.78	4.21E-03
O glycan biosynthesis	27	0.58	1.74	6.86E-03
Aldosterone regulated sodium reabsorption	42	0.52	1.71	8.95E-03
Cardiac muscle contraction	73	0.46	1.69	1.10E-02
Asthma	28	0.54	1.68	1.15E-02
Long term potentiation	69	0.46	1.66	1.44E-02
Amyotrophic lateral sclerosis	52	0.45	1.55	4.17E-02
Nitrogen metabolism	23	0.52	1.54	4.24E-02

Note: Size: Total number of genes in the category; ES: enrichment score; NES: normalized enrichment score; FDR: false discovery rate.

**Table 3:** Basic characteristics of 4 in-house GWAS samples.

Characteristics	KCOS	OOS	COS	CFS	
				Cases	Controls
Sample size	2,286	1,000	1,627	350	350
Population	Caucasian	Caucasian	Han Chinese	Han Chinese	Han Chinese
Female (%)	75.59	50.10	50.71	64.57	50.57
Age (yrs)	51.37 ± 13.76	50.23 ± 18.24	34.49 ± 13.24	69.35 ± 7.41	69.54 ± 6.09
Height (m)	1.66 ± 0.08	1.71 ± 0.10	1.64 ± 0.08	1.63 ± 0.12	1.59 ± 0.10
Weight (kg)	75.27 ± 17.54	80.16 ± 17.79	60.12 ± 10.48	59.15 ± 12.05	59.61 ±10.84
Spine BMD (g/cm^2^)	1.02 ± 0.16	1.03 ± 0.16	0.95 ± 0.13	—	—
Femoral neck BMD (g/cm^2^)	0.80 ± 0.15	0.81 ± 0.14	0.81 ± 0.13	—	—

Notes: Data are shown as mean ± standard deviation.

Abbreviations: KCOS, Kansas-city osteoporosis study; OOS, Omaha osteoporosis study; COS, Chinese osteoporosis study; CFS, Chinese Fractures Study.

**Table 4:** Significant association results between the predicted genes and spine/femoral neck BMD in the GWAS BMD datasets.

SNP	Chr	Position^a^	A1/A2	Genic position	Gene	In-house GWAS samples				GEFOS sample				Combined meta-analysis^b^				*P*_adjusted_
						*P*_FN	Dir	*P*_SP	Dir	*P*_FN	Dir	*P*_SP	Dir	*P*_FN	*P*_SP	*Q*_het_ *P*	*I*^*2*^	
rs7124442	11	27633617	C/T	3′UTR	BDNF	0.265	+	0.074	+	0.082	+	1.26 × 10^−4^	+	0.022	2.47 × 10^−5^	0.75	0	0.02
rs11030119	11	27684678	T/C	intron	BDNF	0.325	+	0.103	+	0.137	+	3.04 × 10^−4^	+	0.041	7.65 × 10^−5^	0.79	0	0.02
rs2938780	5	59132490	C/T	intron	PDE4D	0.586	+	0.080	+	0.004	+	5.35 × 10^−4^	+	0.002	1.15 × 10^−4^	0.67	0	0.02
rs895526	2	199870670	A/G	intron	SATB2	0.269	+	0.212	+	0.784	–	2.84 × 10^−4^	+	0.259	1.25 × 10^−4^	0.91	0	0.02
rs2963826	5	59132680	T/C	intron	PDE4D	0.614	+	0.088	+	0.004	+	5.57 × 10^−4^	+	0.002	1.27 × 10^−4^	0.70	0	0.02
rs6704641	2	199872497	G/A	intron	SATB2	0.357	+	0.203	+	0.806	–	3.04 × 10^−4^	+	0.290	1.28 × 10^−4^	0.94	0	0.02

Note: Chr: chromosome; FN: femoral neck; SP: spine; Dir: direction, which is for the effect of A1 (Minor allele).

^a^Position was relative to the hg18 version of the human genome.

^b^Combined meta-analysis means that the *P* value was combined by including all the GWAS samples. *Q*_het_ is the Cochran’s Q statistic, and *I*^2^ is the measure of heterogeneity.

All *P* values listed in Table 4 are two-sided.

**Table 5:** Association results of the above 6 BMD associated loci from Table 4 with osteoporotic fractures.

SNP	Gene	A1/A2	MAF cases	MAF controls	OR (95%CI)	*P*
rs7124442	BDNF	C/T	0.062	0.089	0.67 (0.45–1.00)	0.042
rs11030119	BDNF	T/C	0.037	0.062	0.58 (0.35–0.96)	0.024
rs2938780	PDE4D	C/T	0.170	0.194	0.85 (0.65–1.11)	0.238
rs895526	SATB2	A/G	0.497	0.491	1.02 (0.83–1.26)	0.825
rs2963826	PDE4D	T/C	0.171	0.194	0.86 (0.65–1.13)	0.268
rs6704641	SATB2	G/A	0.500	0.489	1.04 (0.85–1.29)	0.658

Note: MAF: minor allele frequency for the A1 (minor allele); OR: odds ratio; CI: confidence interval.

**Table 6:** Results of cis-expression quantitative trait locus (eQTL) analyses.

Target SNP	Gene	Surrogate SNPs	*r*^2^, Distance (Kb) to target SNP	*P*_eQTL_
rs7124442	BDNF	rs73432320	1, 7.8	3.06 × 10^−4^
		rs7130131	0.7, 14.2	2.21 × 10^−3^
		rs7927728	0.7, 9.6	2.21 × 10^−3^
rs11030119	BDNF	rs73432320	1, 58.9	3.06 × 10^−4^
		rs7130131	0.7, 65.3	2.21 × 10^−3^
		rs7927728	0.7, 60.6	2.21 × 10^−3^
