# Supplementary material for: Integrating Epigenomic Elements and GWASs Identifies BDNF Gene Affecting Bone Mineral Density and Osteoporotic Fracture Risk
Source: Sci Rep. 2016 Jul 28;6:30558. doi: 10.1038/srep30558 (PMC4964617; doi:10.1038/srep30558)
Supplement: Supplementary Information [file srep30558-s1.pdf]

Integrating Epigenomic Elements and GWASs Identifies *BDNF* Gene Affecting Bone Mineral Density and Osteoporotic Fracture Risk

Yan Guo[1], Shan-Shan Dong[1], Xiao-Feng Chen[1], Ying-Aisha Jing[1], Man Yang[1], Han Yan[1], Hui Shen[2], Xiang-Ding Chen[3], Li-Jun Tan[3], Qing Tian[2], Hong-Wen Deng[2], Tie-Lin Yang[1]*

[1] Key Laboratory of Biomedical Information Engineering of Ministry of Education, School of Life Science and Technology, Xi'an Jiaotong University, Xi'an 710049, P. R. China

[2] School of Public Health and Tropical Medicine, Tulane University

New Orleans, LA 70112, USA

[3] Laboratory of Molecular and Statistical Genetics, College of Life Sciences, Hunan Normal University, Changsha 410081, P. R. China

Supplementary Tables

Table S1. List of osteoporosis-associated genes obtained from the GWAS Catalog and PheGenI database. A total of 259 osteoporosis-associated genes were included.

Table S2. Pathway enrichment analysis for the osteoporosis-associated genes. Pathways with nominal *P* value < 0.05 are shown.

Table S3. Functional enrichment analysis for all the epigenomic elements used in this study. For each element, the -log10 transformed enrichment *P*-values for the osteoporosis-associated gene are shown, along with the *P*-values for the randomly selected genes set of the same size. Epigenomic elements enriched with *P* -value < 0.01 are highlighted in red while depleted epigenomic elements with *P* -value < 0.01 are highlighted in

green.

Table S4. Cell types used to obtain epigenomic elements. Information was obtained from https://genome.ucsc.edu/encode/cellTypes.html.

Table S5. siRNA sequences for *BDNF*.

Table S6. Primer sequences for PCR analyses.

Table S1. List of osteoporosis-associated genes obtained from the GWAS Catalog and PheGenI database.

Gene	Full name	Chromosome	Pathway enriched
ABCF2	ATP-binding cassette, sub-family F (GCN20), member 2	7q36	
ACTL8	actin-like 8	1p36.13	
ADAMTS18	ADAM metallopeptidase with thrombospondin type 1 motif, 18	16q23	
ADGRV1	adhesion G protein-coupled receptor V1	5q13	
ADORA3	adenosine A3 receptor	1p13.2	cGMP-PKG signaling pathway
AKAP11	A kinase (PRKA) anchor protein 11	13q14.11	
AMPH	amphiphysin	7p14-p13	Fc gamma R-mediated phagocytosis
ANAPC1	anaphase promoting complex subunit 1	2q12.1	HTLV-I infection
ARHGAP1	Rho GTPase activating protein 1	11p11.2	
ARHGAP10	Rho GTPase activating protein 10	4q31.23	Bacterial invasion of epithelial cells
ATL2	atlastin GTPase 2	2p22.3	
ATXN3L	ataxin 3-like	Xp22.2	Protein processing in endoplasmic reticulum
AXIN1	axin 1	16p13.3	Wnt signaling pathway
BET1L	Bet1 golgi vesicular membrane trafficking protein-like	11p15.5	SNARE interactions in vesicular transport
BOC	BOC cell adhesion associated, oncogene regulated	3q13.2	
BPIFA3	BPI fold containing family A, member 3	20q11.21	
C10orf90	chromosome 10 open reading frame 90	10q26.2	
C17orf53	chromosome 17 open reading frame 53	17q21.31	
C3orf17	chromosome 3 open reading frame 17	3q13.2	
C6orf10	chromosome 6 open reading frame 10	6p21.3	
CACNA1D	calcium channel, voltage-dependent, L type, alpha 1D subunit	3p14.3	cGMP-PKG signaling pathway
CBR3	carbonyl reductase 3	21q22.2	Metabolic pathways
CCDC158	coiled-coil domain containing 158	4q21.1	
CCDC170	coiled-coil domain containing 170	6q25.1	
CCDC34	coiled-coil domain containing 34	11p14.1	
CD38	CD38 molecule	4p15	Pancreatic secretion
CDC42EP3	CDC42 effector protein (Rho GTPase binding) 3	2p21	
CDH13	cadherin 13	16q23.3	
CDKAL1	CDK5 regulatory subunit associated protein 1-like 1	6p22.3	
CENPW	centromere protein W	6q22.32	
CEP170	centrosomal protein 170kDa	1q44	
CETN3	centrin, EF-hand protein, 3	5q14.3	
CITED2	Cbp/p300-interacting transactivator, with Glu/Asp-rich carboxy-terminal domain, 2	6q23.3	
CKAP5	cytoskeleton associated protein 5	11p11.2	
CLDN14	claudin 14	21q22.3	Leukocyte transendothelial migration
CLEC10A	C-type lectin domain family 10, member A	17p13.1	Cell adhesion molecules (CAMs)

CNTNAP2	contactin associated protein-like 2	7q35	
COL11A1	collagen, type XI, alpha 1	1p21	Focal adhesion
COLEC10	collectin sub-family member 10 (C-type lectin)	8q23-24.1	
COPS8P1	COP9 signalosome subunit 8 pseudogene 1	Xq21.2	
CPED1	cadherin-like and PC-esterase domain containing 1	7q31.31	
CPN1	carboxypeptidase N, polypeptide 1	10q24.2	
CRBN	cereblon	3p26.2	
CRHR1	corticotropin releasing hormone receptor 1	17q21.31	Long-term depression
CRHR1-IT1	CRHR1 intronic transcript 1	17q21.31	
CRYGFP	crystallin, gamma F pseudogene	2q34	
CSMD1	CUB and Sushi multiple domains 1	8p23.2	
CSRNP3	cysteine-serine-rich nuclear protein 3	2q24.3	
CTNNB1	catenin (cadherin-associated protein), beta 1, 88kDa	3p21	Wnt signaling pathway
CTNNBL1	catenin, beta like 1	20q11.23-q11.2	Spliceosome
CYCSP6	cytochrome c, somatic pseudogene 6	2p12	
CYLD	cylindromatosis (turban tumor syndrome)	16q12.1	Osteoclast differentiation
DCDC1	doublecortin domain containing 1	11p13	
DCDC5	doublecortin domain containing 5	11p14.1	
DECR1	2,4-dienoyl CoA reductase 1, mitochondrial	8q21.3	
DGKB	diacylglycerol kinase, beta 90kDa	7p21.2	Metabolic pathways
DHH	desert hedgehog	12q13.1	Hedgehog signaling pathway
DIRAS3	DIRAS family, GTP-binding RAS-like 3	1p31	
DKK1	dickkopf WNT signaling pathway inhibitor 1	10q11.2	Wnt signaling pathway
DNAJC6	DnaJ (Hsp40) homolog, subfamily C, member 6	1p31.3	Endocytosis
DNM3	dynamin 3	1q24.3	Bacterial invasion of epithelial cells
DOCK1	dedicator of cytokinesis 1	10q26.13-q26.3	Bacterial invasion of epithelial cells
DPT	dermatopontin	1q12-q23	
E2F7	E2F transcription factor 7	12q21.2	
EGFL6	EGF-like-domain, multiple 6	Xp22	
EPDR1	ependymin related 1	7p14.1	
ERC1	ELKS/RAB6-interacting/CAST family member 1	12p13.3	NF-kappa B signaling pathway
ERCC4	excision repair cross-complementation group 4	16p13.12	Nucleotide excision repair/Fanconi anemia pathway
ESR1	estrogen receptor 1	6q25.1	Estrogen signaling pathway
EYA4	EYA transcriptional coactivator and phosphatase 4	6q23	
FABP3P2	fatty acid binding protein 3, pseudogene 2	13q13-q14	
FAM155A	family with sequence similarity 155, member A	13q33.3	
FAM174A	family with sequence similarity 174, member A	5q21.1	
FAM210A	family with sequence similarity 210, member A	18p11.21	
FAM3C	family with sequence similarity 3, member C	7q31	
FAM46A	family with sequence similarity 46, member A	6q14	
FAM84A	family with sequence similarity 84, member A	2p24.3	
FAR1	fatty acyl CoA reductase 1	11p15.2	Peroxisome
FBXO15	F-box protein 15	18q22.3	
FGF14	fibroblast growth factor 14	13q34	MAPK signaling pathway

FGFRL1	fibroblast growth factor receptor-like 1	4p16	
FMN2	formin 2	1q43	Dorso-ventral axis formation
FOXL1	forkhead box L1	16q24	
FSIP1	fibrous sheath interacting protein 1	15q14	
FUBP3	far upstream element (FUSE) binding protein 3	9q34.11	
GABPB1	GA binding protein transcription factor, beta subunit 1	15q21.2	
GALNT3	polypeptide N-acetylgalactosaminyltransferase 3	2q24-q31	Metabolic pathways
GPATCH1	G patch domain containing 1	19q13.11	
GRB10	growth factor receptor-bound protein 10	7p12.2	
HAO2	hydroxyacid oxidase 2 (long chain)	1p12	Peroxisome
HDAC5	histone deacetylase 5	17q21	Epstein-Barr virus infection
HDAC9	histone deacetylase 9	7p21.1	
HDGFL1	hepatoma derived growth factor-like 1	6p22.3	
HERPUD2	HERPUD family member 2	7p14.2	
HK2	hexokinase 2	2p13	Type II diabetes mellitus
HLA-E	major histocompatibility complex, class I, E	6p21.3	HTLV-I infection
HMGN2P6	high mobility group nucleosomal binding domain 2 pseudogene 6	14q12	HTLV-I infection
HOXC6	homeobox C6	12q13.3	
HPDL	4-hydroxyphenylpyruvate dioxygenase-like	1p34.1	
HSP90AB3P	heat shock protein 90kDa alpha (cytosolic), class B member 3, pseudogene	4q22.1	
HSPA13	heat shock protein 70kDa family, member 13	21q11	
IBSP	integrin-binding sialoprotein	4q21.1	ECM-receptor interaction
IDUA	iduronidase, alpha-L-	4p16.3	Metabolic pathways
IGSF21	immunoglobin superfamily, member 21	1p36.13	
IL21R	interleukin 21 receptor	16p11	Inflammatory bowel disease (IBD)
INO80	INO80 complex subunit	15q15.1	
INSC	inscuteable homolog (Drosophila)	11p15.2	
INSIG2	insulin induced gene 2	2q14.2	
JAG1	jagged 1	20p12.1-p11.23	Notch signaling pathway
KCNMA1	potassium channel, calcium activated large conductance subfamily M alpha, member 1	10q22.3	cGMP-PKG signaling pathway
KCNS3	potassium voltage-gated channel, modifier subfamily S, member 3	2p24	
KIAA2018	KIAA2018	3q13.2	
KLF6	Kruppel-like factor 6	10p15	
KLHL4	kelch-like family member 4	Xq21.3	
KLHL42	kelch-like family member 42	12p11.22	
LEKR1	leucine, glutamate and lysine rich 1	3q25.31	
LEMD3	LEM domain containing 3	12q14	
LGR4	leucine-rich repeat containing G protein-coupled receptor 4	11p14-p13	
LIN7C	lin-7 homolog C (C. elegans)	11p14	
LOC344382	serine/threonine kinase receptor associated protein pseudogene	2p22.2	
LRP4	low density lipoprotein receptor-related protein 4	11p11.2	
LRP5	low density lipoprotein receptor-related protein 5	11q13.4	

LRRN1	leucine rich repeat neuronal 1	3p26.2	
MAPT	microtubule-associated protein tau	17q21.1	MAPK signaling pathway
11-Mar	membrane-associated ring finger (C3HC4) 11	5p15.1	
MARK3	MAP/microtubule affinity-regulating kinase 3	14q32.32	
MBL2	mannose-binding lectin (protein C) 2, soluble	10q11.2	Phagosome
MCUR1	mitochondrial calcium uniporter regulator 1	6p23	
MEF2C	myocyte enhancer factor 2C	5q14.3	cGMP-PKG signaling pathway
MEPE	matrix extracellular phosphoglycoprotein	4q21.1	cGMP-PKG signaling pathway
MPHOSPH6	M-phase phosphoprotein 6	16q23.3	RNA degradation
MPP7	membrane protein, palmitoylated 7 (MAGUK p55 subfamily member 7)	10p12.1	
MRPL11P3	mitochondrial ribosomal protein L11 pseudogene 3	12q21.2	
MRPS18BP2	mitochondrial ribosomal protein S18B pseudogene 2	2q22.1	
MRPS31P1	mitochondrial ribosomal protein S31 pseudogene 1	3p21.33	
NAV3	neuron navigator 3	12q14.3	
NBAS	neuroblastoma amplified sequence	2p24	
NETO1	neuropilin (NRP) and tolloid (TLL)-like 1	18q22.2	
NME8	NME/NM23 family member 8	7p14.1	
NOTCH4	notch 4	6p21.3	Dorso-ventral axis formation
NR5A2	nuclear receptor subfamily 5, group A, member 2	1q32.1	Maturity onset diabetes of the young
NRSN1	neurensin 1	6p22.3	
NTAN1	N-terminal asparagine amidase	16p13.11	
OMA1	OMA1 zinc metallopeptidase	1p32.2-32.1	
OR2BH1P	olfactory receptor, family 2, subfamily BH, member 1 pseudogene	11p14.1	
OR7E156P	olfactory receptor, family 7, subfamily E, member 156 pseudogene	13q21.31	
OR7K1P	olfactory receptor, family 7, subfamily K, member 1 pseudogene	14q12	
P4HA3	prolyl 4-hydroxylase, alpha polypeptide III	11q13.4	Metabolic pathways
PABPC4L	poly(A) binding protein, cytoplasmic 4-like	4q28.3	RNA degradation
PCDH10	protocadherin 10	4q28.3	
PCNT	pericentrin	21q22.3	
PITRM1	pitrilysin metallopeptidase 1	10p15.2	
PKDCC	protein kinase domain containing, cytoplasmic	2p21	
PKIA	protein kinase (cAMP-dependent, catalytic) inhibitor alpha	8q21.12	Alcoholism
PLCL1	phospholipase C-like 1	2q33	GABAergic synapse
PPIAP7	peptidylprolyl isomerase A (cyclophilin A) pseudogene 7	1p21.2	
PPP6R3	protein phosphatase 6, regulatory subunit 3	11q13	
PRICKLE1	prickle homolog 1	12q12	Wnt signaling pathway
PRKCE	protein kinase C, epsilon	2p21	cGMP-PKG signaling pathway
PRKG2	protein kinase, cGMP-dependent, type II	4q13.1-q21.1	cGMP-PKG signaling pathway
PSMA2	proteasome (prosome, macropain) subunit, alpha type, 2	7p13	cGMP-PKG signaling pathway
PTH	parathyroid hormone	11p15.3-p15.1	
PTH2R	parathyroid hormone 2 receptor	2q33	

PTHLH	parathyroid hormone-like hormone	12p12.1-p11.2	
PTX4	pentraxin 4, long	16p13.3	
RAF1P1	RAF1 pseudogene 1	4p16.1	
RAP1A	RAP1A, member of RAS oncogene family	1p13.3	
RASGEF1B	RasGEF domain family, member 1B	4q21.21	
RBMS3	RNA binding motif, single stranded interacting protein 3	3p24-p23	
RDH14	retinol dehydrogenase 14 (all-trans/9-cis/11-cis)	2p24.2	
REG3G	regenerating islet-derived 3 gamma	2p12	
RERE	arginine-glutamic acid dipeptide (RE) repeats	1p36.23	
RGS8	regulator of G-protein signaling 8	1q25	
RIN3	Ras and Rab interactor 3	14q32.12	
RMDN2	regulator of microtubule dynamics 2	2p22.2	
RNU7-80P	RNA, U7 small nuclear 80 pseudogene	1p31.3	
RORA	RAR-related orphan receptor A	15q22.2	
RPL17P25	ribosomal protein L17 pseudogene 25	6q14.1	
RPL17P44	ribosomal protein L17 pseudogene 44	18q21.33	
RPL19P8	ribosomal protein L19 pseudogene 8	4q34.3	
RPL23AP27	ribosomal protein L23a pseudogene 27	2q12.1	
RPL31P48	ribosomal protein L31 pseudogene 48	12q21.1	
RPL32P28	ribosomal protein L32 pseudogene 28	13q21.31	
RPL36AP25	ribosomal protein L36a pseudogene 25	6p22.3	
RPL39P30	ribosomal protein L39 pseudogene 30	16q24.2	
RPL3P11	ribosomal protein L3 pseudogene 11	9p23	
RPL6P18	ribosomal protein L6 pseudogene 18	6p22.3	
RPL6P2	ribosomal protein L6 pseudogene 2	1p12	
RPL7AP21	ribosomal protein L7a pseudogene 21	1q23	
RPL9P13	ribosomal protein L9 pseudogene 13	2q22.1	
RPLP0P6	ribosomal protein, large, P0 pseudogene 6	2p22.1	
RPS15AP31	ribosomal protein S15a pseudogene 31	11p14.1	
RPS20P25	ribosomal protein S20 pseudogene 25	9q21.31	
RPS26P35	ribosomal protein S26 pseudogene 35	8q24.12	
RPS27P21	ribosomal protein S27 pseudogene 21	12q12	
RPS27P4	ribosomal protein S27 pseudogene 4	3p22.1	
RPS28P7	ribosomal protein S28 pseudogene 7	11q14.1	
RPS3AP24	ribosomal protein S3a pseudogene 24	6q24.1	
RPS4XP9	ribosomal protein S4X pseudogene 9	6q22.32	
RPS6KA5	ribosomal protein S6 kinase, 90kDa, polypeptide 5	14q31-q32.1	MAPK signaling pathway
RPSAP19	ribosomal protein SA pseudogene 19	1p21.1	
RRP15	ribosomal RNA processing 15 homolog (S. cerevisiae)	1q41	
RSL24D1P4	ribosomal L24 domain containing 1 pseudogene 4	1q43	
RSPH14	radial spoke head 14 homolog (Chlamydomonas)	22q11.2	
RSPO3	R-spondin 3	6q22.33	
SALL1	spalt-like transcription factor 1	16q12.1	
SAMSN1	SAM domain, SH3 domain and nuclear localization signals 1	21q11	
SCYL1	SCY1-like 1 (S. cerevisiae)	11q13	

SEMA3C	sema domain, immunoglobulin domain (Ig), short basic domain, secreted, (semaphorin) 3C	7q21-q31	Axon guidance
SHFM1	split hand/foot malformation (ectrodactyly) type 1	7q21.3	Epstein-Barr virus infection
SHISA9	shisa family member 9	16p13.12	
SLC16A11	solute carrier family 16, member 11	11p13.1	
SLC25A13	solute carrier family 25 (aspartate/glutamate carrier), member 13	7q21.3	
SLC4A4	solute carrier family 4 (sodium bicarbonate cotransporter), member 4	4q21	Pancreatic secretion
SMG6	SMG6 nonsense mediated mRNA decay factor	17p13.3	mRNA surveillance pathway
SMOC1	SPARC related modular calcium binding 1	14q24.2	
SOST	sclerostin	17q11.2	Wnt signaling pathway
SOX4	SRY (sex determining region Y)-box 4	6p22.3	MicroRNAs in cancer
SOX6	SRY (sex determining region Y)-box 6	11p15.3	
SOX9	SRY (sex determining region Y)-box 9	17q24.3	
SP1	Sp1 transcription factor	12q13.1	Transcriptional misregulation in cancer
SP7	Sp7 transcription factor	12q13.13	
SPAG16	sperm associated antigen 16	2q34	
SPOCK1	sparc/osteonectin, cwcv and kazal-like domains proteoglycan (testican) 1	5q31.2	
SPTBN1	spectrin, beta, non-erythrocytic 1	2p21	
ST8SIA4	ST8 alpha-N-acetyl-neuraminide alpha-2,8-sialyltransferase 4	5q21	
STARD3NL	STARD3 N-terminal like	7p14-p13	
SUPT3H	suppressor of Ty 3 homolog (S. cerevisiae)	6p21.1-p21.3	Transcriptional misregulation in cancer
TACSTD2	tumor-associated calcium signal transducer 2	1p32.2-32.1	
TANC1	tetratricopeptide repeat, ankyrin repeat and coiled-coil containing 1	2q24.2	
TBX20	T-box 20	7p14.3	
TENM4	teneurin transmembrane protein 4	11q14.1	
TGFBR3	transforming growth factor, beta receptor III	1p33-p32	
TLE1	transducin-like enhancer of split 1 (E(sp1) homolog, Drosophila)	9q21.32	
TMEM135	transmembrane protein 135	11q14.2	
TMEM182	transmembrane protein 182	2q12.1	
TMEM263	transmembrane protein 263	12q23.3	
TNFRSF11A	tumor necrosis factor receptor superfamily, member 11a, NFKB activator	18q22.1	Prolactin signaling pathway
TNFRSF11B	tumor necrosis factor receptor superfamily, member 11b	8q24	Osteoclast differentiation
TNFSF11	tumor necrosis factor (ligand) superfamily, member 11	13q14	Prolactin signaling pathway
TRPC7	transient receptor potential cation channel, subfamily C, member 7	5q31.1	
TSPAN7	tetraspanin 7	Xp11.4	
UBBP2	ubiquitin B pseudogene 2	1q32	
UMOD	uromodulin	16p12.3	
UPK3B	uroplakin 3B	7q11.2	
UQCC1	ubiquinol-cytochrome c reductase complex assembly factor 1	20q11.22	

VENTXP3	VENT homeobox pseudogene 3	12q21.1	
VIT	vitrin	2p22.2	
WHSC1L2P	Wolf-Hirschhorn syndrome candidate 1-like 2, pseudogene	17q21.31	
WIF1	WNT inhibitory factor 1	12q14.3	
WLS	wntless Wnt ligand secretion mediator	1p31.3	
WNT16	wingless-type MMTV integration site family, member 16	7q31	Wnt signaling pathway
WNT4	wingless-type MMTV integration site family, member 4	1p36.23-p35.1	Wnt signaling pathway
XKR9	XK, Kell blood group complex subunit-related family, member 9	8q13.3	
ZBTB40	zinc finger and BTB domain containing 40	1p36	
ZNF408	zinc finger protein 408	11p11.2	
ZNF518B	zinc finger protein 518B	4p16.1	
ZNF622	zinc finger protein 622	5p15.1	
ZSWIM5	zinc finger, SWIM-type containing 5	1p34.1	

Table S2. Pathway enrichment analysis for the known osteoporosis-associated genes.

KEGG ID	Term	Component genes	Enrichment *P*-value	FDR *P*-value
hsa04310	Wnt signaling pathway	DKK1, WIF1, CTNNB1, AXIN1, WNT4, PRICKLE1, WNT16, SOST	8.10E-05	2.33E-02
hsa05217	Basal cell carcinoma	CTNNB1, AXIN1, WNT4, WNT16	2.49E-03	3.57E-01
hsa04022	cGMP-PKG signaling pathway	PRKCE, CACNA1D, PRKG2, KCNMA1, MEF2C, ADORA3	5.84E-03	4.93E-01
hsa05100	Bacterial invasion of epithelial cells	DNM3, CTNNB1, ARHGAP10, DOCK1	6.88E-03	4.93E-01
hsa04930	Type II diabetes mellitus	PRKCE, HK2, CACNA1D	1.12E-02	6.09E-01
hsa04972	Pancreatic secretion	RAP1A, CD38, SLC4A4, KCNMA1	1.41E-02	6.09E-01
hsa04340	Hedgehog signaling pathway	DHH, WNT4, WNT16	1.49E-02	6.09E-01
hsa04320	Dorso-ventral axis formation	FMN2, NOTCH4	2.14E-02	7.66E-01
hsa04919	Thyroid hormone signaling pathway	ESR1, NOTCH4, CTNNB1, WNT4	3.14E-02	9.90E-01
hsa04917	Prolactin signaling pathway	ESR1, TNFRSF11A, TNFSF11	3.45E-02	9.90E-01
hsa04380	Osteoclast differentiation	TNFRSF11B, TNFRSF11A, TNFSF11, CYLD	3.87E-02	1.00E+00
hsa04010	MAPK signaling pathway	CACNA1D, RAP1A, MAPT, MEF2C, RPS6AK5, FGF14	4.62E-02	1.00E+00

Table S3. Functional enrichment analysis for all the epigenomic elements used in this study.

No.	Type	**Cell** line	Epigenomic element	Osteoporosis genes	Random genes
1	Transcription Factor by ChIP-seq	Combined	ARID3A	0.33	-0.52
2	Transcription Factor by ChIP-seq	Combined	ATF1	-0.54	-0.46
3	Transcription Factor by ChIP-seq	Combined	ATF2	0.61	-0.42
4	Transcription Factor by ChIP-seq	Combined	ATF3	-0.98	0.32
5	Transcription Factor by ChIP-seq	Combined	BACH1	-0.53	-0.43
6	Transcription Factor by ChIP-seq	Combined	BATF	0.47	0.66
7	Transcription Factor by ChIP-seq	Combined	BCL11A	0.52	0.36
8	Transcription Factor by ChIP-seq	Combined	BCL3	-0.84	-0.87
9	Transcription Factor by ChIP-seq	Combined	BCLAF1	-0.33	-0.33
10	Transcription Factor by ChIP-seq	Combined	BDP1	0.28	0.53
11	Transcription Factor by ChIP-seq	Combined	BHLHE40	-0.76	-0.36
12	Transcription Factor by ChIP-seq	Combined	BRCA1	-0.43	-0.45
13	Transcription Factor by ChIP-seq	Combined	BRF1	0.29	0.00
14	Transcription Factor by ChIP-seq	Combined	BRF2	0.28	0.13
15	Transcription Factor by ChIP-seq	Combined	CBX3	-0.54	0.46
16	Transcription Factor by ChIP-seq	Combined	CCNT2	-0.53	-0.76
17	Transcription Factor by ChIP-seq	Combined	CEBPB	0.31	-0.37
18	Transcription Factor by ChIP-seq	Combined	CEBPD	-0.40	-0.43
19	Transcription Factor by ChIP-seq	Combined	CHD1	0.38	0.33
20	Transcription Factor by ChIP-seq	Combined	CHD2	-0.35	-0.66
21	Transcription Factor by ChIP-seq	Combined	CREB1	-0.37	-0.69
22	Transcription Factor by ChIP-seq	Combined	CTBP2	0.47	0.96
23	Transcription Factor by ChIP-seq	Combined	CTCF	0.46	-0.41
24	Transcription Factor by ChIP-seq	Combined	CTCFL	-0.88	-0.48
25	Transcription Factor by ChIP-seq	Combined	E2F1	0.37	-0.54
26	Transcription Factor by ChIP-seq	Combined	E2F4	-0.81	-0.39
27	Transcription Factor by ChIP-seq	Combined	E2F6	-0.63	-0.47
28	Transcription Factor by ChIP-seq	Combined	EBF1	-0.45	-0.33
29	Transcription Factor by ChIP-seq	Combined	EGR1	0.32	-0.41
30	Transcription Factor by ChIP-seq	Combined	ELF1	-0.77	-0.66
31	Transcription Factor by ChIP-seq	Combined	ELK1	-1.00	-0.29
32	Transcription Factor by ChIP-seq	Combined	ELK4	-1.86	0.40
33	Transcription Factor by ChIP-seq	Combined	EP300	0.77	0.45
34	Transcription Factor by ChIP-seq	Combined	ESR1	0.28	0.26
35	Transcription Factor by ChIP-seq	Combined	ESRRA	-0.33	0.33
36	Transcription Factor by ChIP-seq	Combined	ETS1	-1.01	-0.42
37	Transcription Factor by ChIP-seq	Combined	EZH2	2.09	-0.47
38	Transcription Factor by ChIP-seq	Combined	FAM48A	-0.14	0.00
39	Transcription Factor by ChIP-seq	Combined	FOS	-0.35	0.48
40	Transcription Factor by ChIP-seq	Combined	FOSL1	-0.38	0.25
41	Transcription Factor by ChIP-seq	Combined	FOSL2	0.39	-0.27
42	Transcription Factor by ChIP-seq	Combined	FOXA1	0.99	-0.33
43	Transcription Factor by ChIP-seq	Combined	FOXA2	0.79	0.34
44	Transcription Factor by ChIP-seq	Combined	FOXM1	0.36	0.37
45	Transcription Factor by ChIP-seq	Combined	FOXP2	0.29	-0.55

46	Transcription Factor by ChIP-seq	Combined	GABPA	-0.92	-0.46
47	Transcription Factor by ChIP-seq	Combined	GATA1	-0.67	0.28
48	Transcription Factor by ChIP-seq	Combined	GATA2	0.45	-0.31
49	Transcription Factor by ChIP-seq	Combined	GATA3	0.90	0.74
50	Transcription Factor by ChIP-seq	Combined	GRp20	-0.66	-0.51
51	Transcription Factor by ChIP-seq	Combined	GTF2B	-0.46	-0.29
52	Transcription Factor by ChIP-seq	Combined	GTF2F1	0.52	-0.44
53	Transcription Factor by ChIP-seq	Combined	GTF3C2	-0.59	0.76
54	Transcription Factor by ChIP-seq	Combined	HDAC1	-1.34	0.29
55	Transcription Factor by ChIP-seq	Combined	HDAC2	0.34	-0.34
56	Transcription Factor by ChIP-seq	Combined	HDAC6	0.00	-0.43
57	Transcription Factor by ChIP-seq	Combined	HDAC8	0.59	0.34
58	Transcription Factor by ChIP-seq	Combined	HMGN3	-0.66	-0.53
59	Transcription Factor by ChIP-seq	Combined	HNF4A	-0.40	-0.49
60	Transcription Factor by ChIP-seq	Combined	HNF4G	-0.29	-0.28
61	Transcription Factor by ChIP-seq	Combined	HSF1	-0.24	-0.39
62	Transcription Factor by ChIP-seq	Combined	IKZF1	0.59	0.86
63	Transcription Factor by ChIP-seq	Combined	IRF1	-1.10	-0.58
64	Transcription Factor by ChIP-seq	Combined	IRF3	0.43	-0.46
65	Transcription Factor by ChIP-seq	Combined	IRF4	0.91	0.44
66	Transcription Factor by ChIP-seq	Combined	JUN	-0.32	0.71
67	Transcription Factor by ChIP-seq	Combined	JUNB	-0.55	1.06
68	Transcription Factor by ChIP-seq	Combined	JUND	0.43	-0.34
69	Transcription Factor by ChIP-seq	Combined	KAP1	0.29	-0.32
70	Transcription Factor by ChIP-seq	Combined	KDM5A	-0.37	-0.70
71	Transcription Factor by ChIP-seq	Combined	KDM5B	-0.62	-0.32
72	Transcription Factor by ChIP-seq	Combined	MAFF	0.91	-0.33
73	Transcription Factor by ChIP-seq	Combined	MAFK	0.75	-0.58
74	Transcription Factor by ChIP-seq	Combined	MAX	-0.40	-0.66
75	Transcription Factor by ChIP-seq	Combined	MAZ	-0.55	-0.40
76	Transcription Factor by ChIP-seq	Combined	MBD4	0.62	0.36
77	Transcription Factor by ChIP-seq	Combined	MEF2A	0.48	0.34
78	Transcription Factor by ChIP-seq	Combined	MEF2C	0.42	0.43
79	Transcription Factor by ChIP-seq	Combined	MTA3	0.35	-0.37
80	Transcription Factor by ChIP-seq	Combined	MXI1	-0.50	-0.43
81	Transcription Factor by ChIP-seq	Combined	MYBL2	0.51	-0.74
82	Transcription Factor by ChIP-seq	Combined	MYC	-0.53	-0.45
83	Transcription Factor by ChIP-seq	Combined	NANOG	0.68	-0.51
84	Transcription Factor by ChIP-seq	Combined	NFATC1	0.29	0.30
85	Transcription Factor by ChIP-seq	Combined	NFE2	-0.63	-0.49
86	Transcription Factor by ChIP-seq	Combined	NFIC	-0.55	-0.39
87	Transcription Factor by ChIP-seq	Combined	NFYA	-0.30	-1.06
88	Transcription Factor by ChIP-seq	Combined	NFYB	0.37	-0.59
89	Transcription Factor by ChIP-seq	Combined	NR2C2	-0.46	-0.54
90	Transcription Factor by ChIP-seq	Combined	NR2F2	-0.89	0.56
91	Transcription Factor by ChIP-seq	Combined	NR3C1	0.43	-0.42
92	Transcription Factor by ChIP-seq	Combined	NRF1	-0.55	-0.36
93	Transcription Factor by ChIP-seq	Combined	PAX5	0.52	-0.50

94	Transcription Factor by ChIP-seq	Combined	PBX3	0.29	-0.71
95	Transcription Factor by ChIP-seq	Combined	PHF8	-0.68	-0.39
96	Transcription Factor by ChIP-seq	Combined	PML	-0.40	-0.44
97	Transcription Factor by ChIP-seq	Combined	POLR2A	0.44	-0.43
98	Transcription Factor by ChIP-seq	Combined	POLR3G	-0.14	0.00
99	Transcription Factor by ChIP-seq	Combined	POU2F2	0.32	0.48
100	Transcription Factor by ChIP-seq	Combined	POU5F1	0.69	0.48
101	Transcription Factor by ChIP-seq	Combined	PPARGC1A	-0.22	-0.27
102	Transcription Factor by ChIP-seq	Combined	PRDM1	0.26	-0.41
103	Transcription Factor by ChIP-seq	Combined	RAD21	1.48	-0.60
104	Transcription Factor by ChIP-seq	Combined	RBBP5	0.74	-0.48
105	Transcription Factor by ChIP-seq	Combined	RCOR1	-0.40	-0.29
106	Transcription Factor by ChIP-seq	Combined	RDBP	0.26	0.46
107	Transcription Factor by ChIP-seq	Combined	RELA	-0.34	-0.35
108	Transcription Factor by ChIP-seq	Combined	REST	0.31	-0.35
109	Transcription Factor by ChIP-seq	Combined	RFX5	0.48	-0.94
110	Transcription Factor by ChIP-seq	Combined	RPC155	-0.34	0.77
111	Transcription Factor by ChIP-seq	Combined	RUNX3	0.28	0.29
112	Transcription Factor by ChIP-seq	Combined	RXRA	-0.41	0.29
113	Transcription Factor by ChIP-seq	Combined	SAP30	-0.55	-0.40
114	Transcription Factor by ChIP-seq	Combined	SETDB1	0.43	-0.29
115	Transcription Factor by ChIP-seq	Combined	SIN3A	0.38	-0.93
116	Transcription Factor by ChIP-seq	Combined	SIN3AK20	0.48	-0.32
117	Transcription Factor by ChIP-seq	Combined	SIRT6	-0.33	0.33
118	Transcription Factor by ChIP-seq	Combined	SIX5	-0.66	-0.91
119	Transcription Factor by ChIP-seq	Combined	SMARCA4	0.62	-0.47
120	Transcription Factor by ChIP-seq	Combined	SMARCB1	0.65	-0.42
121	Transcription Factor by ChIP-seq	Combined	SMARCC1	0.27	-0.88
122	Transcription Factor by ChIP-seq	Combined	SMARCC2	0.49	-0.75
123	Transcription Factor by ChIP-seq	Combined	SMC3	-0.84	-0.50
124	Transcription Factor by ChIP-seq	Combined	SP1	0.60	-0.74
125	Transcription Factor by ChIP-seq	Combined	SP2	-0.27	-0.86
126	Transcription Factor by ChIP-seq	Combined	SP4	-0.48	0.45
127	Transcription Factor by ChIP-seq	Combined	SPI1	-0.51	-0.28
128	Transcription Factor by ChIP-seq	Combined	SREBP1	-0.32	-0.29
129	Transcription Factor by ChIP-seq	Combined	SRF	-0.72	-0.51
130	Transcription Factor by ChIP-seq	Combined	STAT1	-0.49	-0.53
131	Transcription Factor by ChIP-seq	Combined	STAT2	-0.36	0.43
132	Transcription Factor by ChIP-seq	Combined	STAT3	-0.60	0.42
133	Transcription Factor by ChIP-seq	Combined	STAT5A	-0.26	0.62
134	Transcription Factor by ChIP-seq	Combined	SUZ12	0.93	-0.26
135	Transcription Factor by ChIP-seq	Combined	TAF1	0.68	-0.38
136	Transcription Factor by ChIP-seq	Combined	TAF7	-0.51	-0.73
137	Transcription Factor by ChIP-seq	Combined	TAL1	0.37	0.38
138	Transcription Factor by ChIP-seq	Combined	TBL1XR1	-0.71	-0.29
139	Transcription Factor by ChIP-seq	Combined	TBP	0.38	-0.40
140	Transcription Factor by ChIP-seq	Combined	TCF12	1.16	0.29
141	Transcription Factor by ChIP-seq	Combined	TCF3	-0.28	0.33

142	Transcription Factor by ChIP-seq	Combined	TCF7L2	0.35	-0.77
143	Transcription Factor by ChIP-seq	Combined	TEAD4	-0.51	-0.30
144	Transcription Factor by ChIP-seq	Combined	TFAP2A	-0.54	-1.34
145	Transcription Factor by ChIP-seq	Combined	TFAP2C	-0.60	-0.59
146	Transcription Factor by ChIP-seq	Combined	THAP1	-0.46	-0.57
147	Transcription Factor by ChIP-seq	Combined	TRIM28	0.46	0.63
148	Transcription Factor by ChIP-seq	Combined	UBTF	-0.42	-0.42
149	Transcription Factor by ChIP-seq	Combined	USF1	-0.34	-0.35
150	Transcription Factor by ChIP-seq	Combined	USF2	0.43	-0.42
151	Transcription Factor by ChIP-seq	Combined	WRNIP1	0.28	-0.43
152	Transcription Factor by ChIP-seq	Combined	YY1	0.48	-0.40
153	Transcription Factor by ChIP-seq	Combined	ZBTB33	-0.35	-0.61
154	Transcription Factor by ChIP-seq	Combined	ZBTB7A	-1.06	-0.40
155	Transcription Factor by ChIP-seq	Combined	ZEB1	-0.33	-0.34
156	Transcription Factor by ChIP-seq	Combined	ZKSCAN1	-0.26	0.43
157	Transcription Factor by ChIP-seq	Combined	ZNF143	0.39	-0.63
158	Transcription Factor by ChIP-seq	Combined	ZNF217	1.02	0.24
159	Transcription Factor by ChIP-seq	Combined	ZNF263	0.52	-0.28
160	Transcription Factor by ChIP-seq	Combined	ZNF274	-0.14	0.18
161	Transcription Factor by ChIP-seq	Combined	ZZZ3	-0.18	0.50
162	Chromatin State Segmentation by HMM	Gm12878	1_Active_Promoter	-0.67	0.50
163	Chromatin State Segmentation by HMM	Gm12878	10_Txn_Elongation	-1.46	-0.26
164	Chromatin State Segmentation by HMM	Gm12878	11_Weak_Txn	-0.99	0.42
165	Chromatin State Segmentation by HMM	Gm12878	12_Repressed	2.72	0.37
166	Chromatin State Segmentation by HMM	Gm12878	13_Heterochrom/lo	-0.36	0.95
167	Chromatin State Segmentation by HMM	Gm12878	14_Repetitive/CNV	0.27	0.69
168	Chromatin State Segmentation by HMM	Gm12878	15_Repetitive/CNV	-0.14	0.00
169	Chromatin State Segmentation by HMM	Gm12878	2_Weak_Promoter	-0.58	-0.34
170	Chromatin State Segmentation by HMM	Gm12878	3_Poised_Promoter	2.22	-0.29
171	Chromatin State Segmentation by HMM	Gm12878	4_Strong_Enhancer	-0.41	0.50
172	Chromatin State Segmentation by HMM	Gm12878	5_Strong_Enhancer	-0.31	0.51
173	Chromatin State Segmentation by HMM	Gm12878	6_Weak_Enhancer	-0.54	-0.48
174	Chromatin State Segmentation by HMM	Gm12878	7_Weak_Enhancer	-0.44	0.41
175	Chromatin State Segmentation by HMM	Gm12878	8_Insulator	0.44	0.36
176	Chromatin State Segmentation by HMM	Gm12878	9_Txn_Transition	0.00	0.34
177	Chromatin State Segmentation by HMM	H1hesc	1_Active_Promoter	-0.49	0.39
178	Chromatin State Segmentation by HMM	H1hesc	10_Txn_Elongation	-1.37	-0.34
179	Chromatin State Segmentation by HMM	H1hesc	11_Weak_Txn	-2.52	-0.47
180	Chromatin State Segmentation by HMM	H1hesc	12_Repressed	2.22	-0.32
181	Chromatin State Segmentation by HMM	H1hesc	13_Heterochrom/lo	0.33	1.23
182	Chromatin State Segmentation by HMM	H1hesc	14_Repetitive/CNV	-0.14	0.00
183	Chromatin State Segmentation by HMM	H1hesc	15_Repetitive/CNV	0.00	0.00
184	Chromatin State Segmentation by HMM	H1hesc	2_Weak_Promoter	-0.41	0.40
185	Chromatin State Segmentation by HMM	H1hesc	3_Poised_Promoter	3.07	0.37
186	Chromatin State Segmentation by HMM	H1hesc	4_Strong_Enhancer	-0.46	-0.34
187	Chromatin State Segmentation by HMM	H1hesc	5_Strong_Enhancer	-0.35	-0.66
188	Chromatin State Segmentation by HMM	H1hesc	6_Weak_Enhancer	-0.77	0.37
189	Chromatin State Segmentation by HMM	H1hesc	7_Weak_Enhancer	-0.80	-0.39

190	Chromatin State Segmentation by HMM	H1hesc	8_Insulator	0.29	-0.30
191	Chromatin State Segmentation by HMM	H1hesc	9_Txn_Transition	0.00	-0.45
192	Chromatin State Segmentation by HMM	Hepg2	1_Active_Promoter	-0.91	0.30
193	Chromatin State Segmentation by HMM	Hepg2	10_Txn_Elongation	-2.10	0.73
194	Chromatin State Segmentation by HMM	Hepg2	11_Weak_Txn	-3.19	-0.28
195	Chromatin State Segmentation by HMM	Hepg2	12_Repressed	0.98	-1.10
196	Chromatin State Segmentation by HMM	Hepg2	13_Heterochrom/lo	0.55	1.28
197	Chromatin State Segmentation by HMM	Hepg2	14_Repetitive/CNV	0.28	0.32
198	Chromatin State Segmentation by HMM	Hepg2	15_Repetitive/CNV	0.00	0.00
199	Chromatin State Segmentation by HMM	Hepg2	2_Weak_Promoter	-0.61	-0.32
200	Chromatin State Segmentation by HMM	Hepg2	3_Poised_Promoter	1.04	-0.36
201	Chromatin State Segmentation by HMM	Hepg2	4_Strong_Enhancer	-0.61	-0.32
202	Chromatin State Segmentation by HMM	Hepg2	5_Strong_Enhancer	0.31	-0.99
203	Chromatin State Segmentation by HMM	Hepg2	6_Weak_Enhancer	-0.63	0.41
204	Chromatin State Segmentation by HMM	Hepg2	7_Weak_Enhancer	-0.68	0.97
205	Chromatin State Segmentation by HMM	Hepg2	8_Insulator	0.32	0.53
206	Chromatin State Segmentation by HMM	Hepg2	9_Txn_Transition	-1.83	0.44
207	Chromatin State Segmentation by HMM	Hmec	1_Active_Promoter	-0.55	0.35
208	Chromatin State Segmentation by HMM	Hmec	10_Txn_Elongation	-1.61	-0.90
209	Chromatin State Segmentation by HMM	Hmec	11_Weak_Txn	-1.78	0.68
210	Chromatin State Segmentation by HMM	Hmec	12_Repressed	1.79	-0.37
211	Chromatin State Segmentation by HMM	Hmec	13_Heterochrom/lo	0.32	0.59
212	Chromatin State Segmentation by HMM	Hmec	14_Repetitive/CNV	0.00	0.00
213	Chromatin State Segmentation by HMM	Hmec	15_Repetitive/CNV	0.00	0.00
214	Chromatin State Segmentation by HMM	Hmec	2_Weak_Promoter	-0.31	0.28
215	Chromatin State Segmentation by HMM	Hmec	3_Poised_Promoter	2.08	0.43
216	Chromatin State Segmentation by HMM	Hmec	4_Strong_Enhancer	-0.32	0.46
217	Chromatin State Segmentation by HMM	Hmec	5_Strong_Enhancer	-0.68	0.30
218	Chromatin State Segmentation by HMM	Hmec	6_Weak_Enhancer	0.32	0.30
219	Chromatin State Segmentation by HMM	Hmec	7_Weak_Enhancer	-1.07	0.75
220	Chromatin State Segmentation by HMM	Hmec	8_Insulator	-0.28	0.73
221	Chromatin State Segmentation by HMM	Hmec	9_Txn_Transition	-1.29	-0.27
222	Chromatin State Segmentation by HMM	Hsmm	1_Active_Promoter	0.34	0.60
223	Chromatin State Segmentation by HMM	Hsmm	10_Txn_Elongation	-1.44	-0.36
224	Chromatin State Segmentation by HMM	Hsmm	11_Weak_Txn	0.28	0.45
225	Chromatin State Segmentation by HMM	Hsmm	12_Repressed	0.77	-0.67
226	Chromatin State Segmentation by HMM	Hsmm	13_Heterochrom/lo	-0.45	1.05
227	Chromatin State Segmentation by HMM	Hsmm	14_Repetitive/CNV	-0.33	0.20
228	Chromatin State Segmentation by HMM	Hsmm	15_Repetitive/CNV	0.00	0.00
229	Chromatin State Segmentation by HMM	Hsmm	2_Weak_Promoter	0.41	0.32
230	Chromatin State Segmentation by HMM	Hsmm	3_Poised_Promoter	1.18	-0.71
231	Chromatin State Segmentation by HMM	Hsmm	4_Strong_Enhancer	-0.27	0.75
232	Chromatin State Segmentation by HMM	Hsmm	5_Strong_Enhancer	0.33	0.31
233	Chromatin State Segmentation by HMM	Hsmm	6_Weak_Enhancer	0.41	0.72
234	Chromatin State Segmentation by HMM	Hsmm	7_Weak_Enhancer	-0.85	0.50
235	Chromatin State Segmentation by HMM	Hsmm	8_Insulator	0.97	-0.25
236	Chromatin State Segmentation by HMM	Hsmm	9_Txn_Transition	-1.28	-0.25
237	Chromatin State Segmentation by HMM	Huvec	1_Active_Promoter	-0.31	0.30

238	Chromatin State Segmentation by HMM	Huvec	10_Txn_Elongation	-1.25	-0.34
239	Chromatin State Segmentation by HMM	Huvec	11_Weak_Txn	-0.90	-0.42
240	Chromatin State Segmentation by HMM	Huvec	12_Repressed	1.30	0.46
241	Chromatin State Segmentation by HMM	Huvec	13_Heterochrom/lo	-0.86	0.76
242	Chromatin State Segmentation by HMM	Huvec	14_Repetitive/CNV	0.00	0.18
243	Chromatin State Segmentation by HMM	Huvec	15_Repetitive/CNV	-0.33	0.00
244	Chromatin State Segmentation by HMM	Huvec	2_Weak_Promoter	0.32	0.36
245	Chromatin State Segmentation by HMM	Huvec	3_Poised_Promoter	0.77	-0.39
246	Chromatin State Segmentation by HMM	Huvec	4_Strong_Enhancer	-0.28	0.42
247	Chromatin State Segmentation by HMM	Huvec	5_Strong_Enhancer	0.26	-0.28
248	Chromatin State Segmentation by HMM	Huvec	6_Weak_Enhancer	0.30	-0.30
249	Chromatin State Segmentation by HMM	Huvec	7_Weak_Enhancer	-0.43	0.36
250	Chromatin State Segmentation by HMM	Huvec	8_Insulator	-0.30	0.36
251	Chromatin State Segmentation by HMM	Huvec	9_Txn_Transition	-0.66	0.25
252	Chromatin State Segmentation by HMM	K562	1_Active_Promoter	-0.91	0.28
253	Chromatin State Segmentation by HMM	K562	10_Txn_Elongation	-1.25	-0.26
254	Chromatin State Segmentation by HMM	K562	11_Weak_Txn	-0.89	-0.33
255	Chromatin State Segmentation by HMM	K562	12_Repressed	0.52	-0.46
256	Chromatin State Segmentation by HMM	K562	13_Heterochrom/lo	0.55	1.02
257	Chromatin State Segmentation by HMM	K562	14_Repetitive/CNV	0.27	-0.28
258	Chromatin State Segmentation by HMM	K562	15_Repetitive/CNV	-0.21	-0.28
259	Chromatin State Segmentation by HMM	K562	2_Weak_Promoter	-0.29	-0.52
260	Chromatin State Segmentation by HMM	K562	3_Poised_Promoter	-0.47	-0.97
261	Chromatin State Segmentation by HMM	K562	4_Strong_Enhancer	-0.98	0.43
262	Chromatin State Segmentation by HMM	K562	5_Strong_Enhancer	-1.37	0.44
263	Chromatin State Segmentation by HMM	K562	6_Weak_Enhancer	0.28	-0.40
264	Chromatin State Segmentation by HMM	K562	7_Weak_Enhancer	-1.11	0.43
265	Chromatin State Segmentation by HMM	K562	8_Insulator	0.34	0.58
266	Chromatin State Segmentation by HMM	K562	9_Txn_Transition	-1.04	-1.14
267	Chromatin State Segmentation by HMM	Nhek	1_Active_Promoter	-0.35	0.36
268	Chromatin State Segmentation by HMM	Nhek	10_Txn_Elongation	-2.15	-0.84
269	Chromatin State Segmentation by HMM	Nhek	11_Weak_Txn	-1.18	0.28
270	Chromatin State Segmentation by HMM	Nhek	12_Repressed	1.24	-0.53
271	Chromatin State Segmentation by HMM	Nhek	13_Heterochrom/lo	-0.30	1.05
272	Chromatin State Segmentation by HMM	Nhek	14_Repetitive/CNV	0.00	0.32
273	Chromatin State Segmentation by HMM	Nhek	15_Repetitive/CNV	0.00	0.00
274	Chromatin State Segmentation by HMM	Nhek	2_Weak_Promoter	-0.30	-0.36
275	Chromatin State Segmentation by HMM	Nhek	3_Poised_Promoter	1.70	-0.79
276	Chromatin State Segmentation by HMM	Nhek	4_Strong_Enhancer	-0.32	0.46
277	Chromatin State Segmentation by HMM	Nhek	5_Strong_Enhancer	-0.79	0.38
278	Chromatin State Segmentation by HMM	Nhek	6_Weak_Enhancer	-0.28	0.36
279	Chromatin State Segmentation by HMM	Nhek	7_Weak_Enhancer	-0.39	0.50
280	Chromatin State Segmentation by HMM	Nhek	8_Insulator	0.42	0.59
281	Chromatin State Segmentation by HMM	Nhek	9_Txn_Transition	-1.83	0.27
282	Chromatin State Segmentation by HMM	Nhlf	1_Active_Promoter	-0.40	0.57
283	Chromatin State Segmentation by HMM	Nhlf	10_Txn_Elongation	-2.63	-0.25
284	Chromatin State Segmentation by HMM	Nhlf	11_Weak_Txn	-0.43	0.55
285	Chromatin State Segmentation by HMM	Nhlf	12_Repressed	1.23	0.34

286	Chromatin State Segmentation by HMM	Nhlf	13_Heterochrom/lo	-0.42	0.80
287	Chromatin State Segmentation by HMM	Nhlf	14_Repetitive/CNV	0.00	0.13
288	Chromatin State Segmentation by HMM	Nhlf	15_Repetitive/CNV	0.00	0.00
289	Chromatin State Segmentation by HMM	Nhlf	2_Weak_Promoter	0.78	-0.38
290	Chromatin State Segmentation by HMM	Nhlf	3_Poised_Promoter	1.18	-0.60
291	Chromatin State Segmentation by HMM	Nhlf	4_Strong_Enhancer	-0.68	0.30
292	Chromatin State Segmentation by HMM	Nhlf	5_Strong_Enhancer	-1.31	-0.37
293	Chromatin State Segmentation by HMM	Nhlf	6_Weak_Enhancer	0.94	0.28
294	Chromatin State Segmentation by HMM	Nhlf	7_Weak_Enhancer	-0.42	-0.37
295	Chromatin State Segmentation by HMM	Nhlf	8_Insulator	0.45	0.38
296	Chromatin State Segmentation by HMM	Nhlf	9_Txn_Transition	-0.80	0.34
297	Histone Modifications by ChIP-seq	A549	CTCF	-0.39	-0.66
298	Histone Modifications by ChIP-seq	A549	H2A.Z	0.82	0.34
299	Histone Modifications by ChIP-seq	A549	H3K27ac	0.34	-0.74
300	Histone Modifications by ChIP-seq	A549	H3K27me3	2.38	0.33
301	Histone Modifications by ChIP-seq	A549	H3K36me3	-0.81	-0.34
302	Histone Modifications by ChIP-seq	A549	H3K4me1	0.31	-0.59
303	Histone Modifications by ChIP-seq	A549	H3K4me2	0.67	-0.35
304	Histone Modifications by ChIP-seq	A549	H3K4me3	0.69	-0.43
305	Histone Modifications by ChIP-seq	A549	H3K79me2	-0.41	-0.43
306	Histone Modifications by ChIP-seq	A549	CTCF	0.46	-0.59
307	Histone Modifications by ChIP-seq	A549	H2A.Z	0.68	-0.31
308	Histone Modifications by ChIP-seq	A549	H3K27ac	-0.42	-0.34
309	Histone Modifications by ChIP-seq	A549	H3K27me3	2.08	-0.42
310	Histone Modifications by ChIP-seq	A549	H3K36me3	-1.00	0.30
311	Histone Modifications by ChIP-seq	A549	H3K4me1	0.43	-0.49
312	Histone Modifications by ChIP-seq	A549	H3K4me2	0.60	-0.37
313	Histone Modifications by ChIP-seq	A549	H3K4me3	0.73	0.29
314	Histone Modifications by ChIP-seq	A549	H3K79me2	-0.39	-0.54
315	Histone Modifications by ChIP-seq	A549	H3K9ac	0.42	-0.45
316	Histone Modifications by ChIP-seq	A549	H3K9me3	0.84	0.70
317	Histone Modifications by ChIP-seq	A549	H4K20me1	-0.30	-0.38
318	Histone Modifications by ChIP-seq	CD20+	CTCF	-0.29	-0.56
319	Histone Modifications by ChIP-seq	CD20+	H2A.Z	0.38	-0.38
320	Histone Modifications by ChIP-seq	CD20+	H3K4me2	0.51	0.35
321	Histone Modifications by ChIP-seq	CD20+_RO01794	EZH2_(39875)	1.55	0.64
322	Histone Modifications by ChIP-seq	CD20+_RO01794	H3K27ac	-0.53	0.37
323	Histone Modifications by ChIP-seq	CD20+_RO01794	H4K20me1	-0.60	-0.42
324	Histone Modifications by ChIP-seq	Dnd41	CTCF	-0.60	-0.36
325	Histone Modifications by ChIP-seq	Dnd41	EZH2_(39875)	0.46	0.30
326	Histone Modifications by ChIP-seq	Dnd41	H2A.Z	-0.50	0.59
327	Histone Modifications by ChIP-seq	Dnd41	H3K27ac	-1.39	0.45
328	Histone Modifications by ChIP-seq	Dnd41	H3K27me3	1.10	0.30
329	Histone Modifications by ChIP-seq	Dnd41	H3K36me3	-2.23	0.39
330	Histone Modifications by ChIP-seq	Dnd41	H3K4me1	-0.82	0.37
331	Histone Modifications by ChIP-seq	Dnd41	H3K4me2	-0.58	-0.35
332	Histone Modifications by ChIP-seq	Dnd41	H3K4me3	-0.51	-0.36
333	Histone Modifications by ChIP-seq	Dnd41	H3K79me2	-1.19	0.37

334	Histone Modifications by ChIP-seq	Dnd41	H3K9ac	-0.69	-0.29
335	Histone Modifications by ChIP-seq	Dnd41	H3K9me3	0.59	0.55
336	Histone Modifications by ChIP-seq	Dnd41	H4K20me1	-1.15	0.31
337	Histone Modifications by ChIP-seq	GM12878	CTCF	-0.35	-0.61
338	Histone Modifications by ChIP-seq	GM12878	EZH2_(39875)	1.48	0.63
339	Histone Modifications by ChIP-seq	GM12878	H2A.Z	0.33	-0.42
340	Histone Modifications by ChIP-seq	GM12878	H3K27ac	-0.52	0.39
341	Histone Modifications by ChIP-seq	GM12878	H3K27me3	1.36	0.46
342	Histone Modifications by ChIP-seq	GM12878	H3K36me3	-1.56	0.33
343	Histone Modifications by ChIP-seq	GM12878	H3K4me1	-0.33	-0.29
344	Histone Modifications by ChIP-seq	GM12878	H3K4me2	0.39	-0.35
345	Histone Modifications by ChIP-seq	GM12878	H3K4me3	0.33	-0.33
346	Histone Modifications by ChIP-seq	GM12878	H3K79me2	-0.57	0.48
347	Histone Modifications by ChIP-seq	GM12878	H3K9ac	-0.39	-0.32
348	Histone Modifications by ChIP-seq	GM12878	H3K9me3	0.76	0.51
349	Histone Modifications by ChIP-seq	GM12878	H4K20me1	-0.73	-0.29
350	Histone Modifications by ChIP-seq	H1hesc	CHD1_(A301-218A)	0.49	0.30
351	Histone Modifications by ChIP-seq	H1hesc	CHD7_(A301-223A-1)	0.61	0.33
352	Histone Modifications by ChIP-seq	H1hesc	CTCF	0.31	-0.53
353	Histone Modifications by ChIP-seq	H1hesc	EZH2_(39875)	0.69	-0.30
354	Histone Modifications by ChIP-seq	H1hesc	H2A.Z	1.05	0.60
355	Histone Modifications by ChIP-seq	H1hesc	H3K27ac	-0.78	-0.29
356	Histone Modifications by ChIP-seq	H1hesc	H3K27me3	1.99	0.30
357	Histone Modifications by ChIP-seq	H1hesc	H3K36me3	-1.47	-0.28
358	Histone Modifications by ChIP-seq	H1hesc	H3K4me1	0.48	-0.37
359	Histone Modifications by ChIP-seq	H1hesc	H3K4me2	0.66	0.35
360	Histone Modifications by ChIP-seq	H1hesc	H3K4me3	0.87	-0.35
361	Histone Modifications by ChIP-seq	H1hesc	H3K79me2	-0.28	-0.31
362	Histone Modifications by ChIP-seq	H1hesc	H3K9ac	-0.36	-0.32
363	Histone Modifications by ChIP-seq	H1hesc	H3K9me3	-0.53	0.40
364	Histone Modifications by ChIP-seq	H1hesc	H4K20me1	0.37	0.37
365	Histone Modifications by ChIP-seq	H1hesc	HDAC2_(A300-705A)	0.68	-0.48
366	Histone Modifications by ChIP-seq	H1hesc	HDAC6_(A301-341A)	-0.64	-0.52
367	Histone Modifications by ChIP-seq	H1hesc	JARID1A_(ab26049)	0.34	0.40
368	Histone Modifications by ChIP-seq	H1hesc	JMJD2A_(A300-861A-1)	0.76	0.32
369	Histone Modifications by ChIP-seq	H1hesc	P300_KAT3B	-0.38	-0.35
370	Histone Modifications by ChIP-seq	H1hesc	PHF8_(A301-772A)	0.32	-0.51
371	Histone Modifications by ChIP-seq	H1hesc	PLU1	-0.71	-0.29
372	Histone Modifications by ChIP-seq	H1hesc	RBBP5_(A300-109A)	0.44	0.30
373	Histone Modifications by ChIP-seq	H1hesc	SAP30_(39731)	0.55	-0.56
374	Histone Modifications by ChIP-seq	H1hesc	SIRT6	-0.57	0.31
375	Histone Modifications by ChIP-seq	H1hesc	SUZ12_(05-1317)	0.83	0.44
376	Histone Modifications by ChIP-seq	HeLa-S3	CTCF	-0.51	0.50
377	Histone Modifications by ChIP-seq	HeLa-S3	EZH2_(39875)	0.84	-0.57
378	Histone Modifications by ChIP-seq	HeLa-S3	H2A.Z	0.44	0.48
379	Histone Modifications by ChIP-seq	HeLa-S3	H3K27ac	-0.62	-0.33
380	Histone Modifications by ChIP-seq	HeLa-S3	H3K27me3	0.83	-0.53
381	Histone Modifications by ChIP-seq	HeLa-S3	H3K36me3	-0.59	0.54

382	Histone Modifications by ChIP-seq	HeLa-S3	H3K4me1	-0.53	0.47
383	Histone Modifications by ChIP-seq	HeLa-S3	H3K4me2	-0.48	0.48
384	Histone Modifications by ChIP-seq	HeLa-S3	H3K4me3	-0.42	0.34
385	Histone Modifications by ChIP-seq	HeLa-S3	H3K79me2	-0.68	0.33
386	Histone Modifications by ChIP-seq	HeLa-S3	H3K9ac	-0.82	-0.31
387	Histone Modifications by ChIP-seq	HeLa-S3	H3K9me3	1.12	0.28
388	Histone Modifications by ChIP-seq	HeLa-S3	H4K20me1	-0.28	0.50
389	Histone Modifications by ChIP-seq	HepG2	CTCF	-0.96	-0.36
390	Histone Modifications by ChIP-seq	HepG2	EZH2_(39875)	0.68	-0.32
391	Histone Modifications by ChIP-seq	HepG2	H2A.Z	0.82	0.36
392	Histone Modifications by ChIP-seq	HepG2	H3K27ac	-1.01	-0.39
393	Histone Modifications by ChIP-seq	HepG2	H3K27me3	0.38	0.29
394	Histone Modifications by ChIP-seq	HepG2	H3K36me3	-1.53	0.32
395	Histone Modifications by ChIP-seq	HepG2	H3K4me1	-0.46	0.31
396	Histone Modifications by ChIP-seq	HepG2	H3K4me2	-0.38	-0.36
397	Histone Modifications by ChIP-seq	HepG2	H3K4me3	-0.44	-0.45
398	Histone Modifications by ChIP-seq	HepG2	H3K79me2	-0.81	0.32
399	Histone Modifications by ChIP-seq	HepG2	H3K9ac	-1.15	0.31
400	Histone Modifications by ChIP-seq	HepG2	H3K9me3	-1.50	0.37
401	Histone Modifications by ChIP-seq	HepG2	H4K20me1	-0.45	0.50
402	Histone Modifications by ChIP-seq	Hmec	CTCF	0.98	0.55
403	Histone Modifications by ChIP-seq	Hmec	EZH2_(39875)	-0.46	-0.33
404	Histone Modifications by ChIP-seq	Hmec	H2A.Z	0.60	-0.35
405	Histone Modifications by ChIP-seq	Hmec	H3K27ac	-0.41	0.30
406	Histone Modifications by ChIP-seq	Hmec	H3K27me3	1.55	-0.33
407	Histone Modifications by ChIP-seq	Hmec	H3K36me3	-0.72	0.33
408	Histone Modifications by ChIP-seq	Hmec	H3K4me1	0.46	-0.40
409	Histone Modifications by ChIP-seq	Hmec	H3K4me2	0.66	0.37
410	Histone Modifications by ChIP-seq	Hmec	H3K4me3	0.61	-0.40
411	Histone Modifications by ChIP-seq	Hmec	H3K79me2	-0.51	0.33
412	Histone Modifications by ChIP-seq	Hmec	H3K9ac	0.28	0.40
413	Histone Modifications by ChIP-seq	Hmec	H3K9me3	-0.53	0.34
414	Histone Modifications by ChIP-seq	Hmec	H4K20me1	0.96	-0.29
415	Histone Modifications by ChIP-seq	Hsmm	CTCF	0.34	-0.56
416	Histone Modifications by ChIP-seq	Hsmm	EZH2_(39875)	1.91	0.29
417	Histone Modifications by ChIP-seq	Hsmm	H2A.Z	0.59	-0.30
418	Histone Modifications by ChIP-seq	Hsmm	H3K27ac	-0.33	-0.33
419	Histone Modifications by ChIP-seq	Hsmm	H3K27me3	1.22	-0.29
420	Histone Modifications by ChIP-seq	Hsmm	H3K36me3	-0.91	0.62
421	Histone Modifications by ChIP-seq	Hsmm	H3K4me1	-0.37	-0.31
422	Histone Modifications by ChIP-seq	Hsmm	H3K4me2	0.59	-0.33
423	Histone Modifications by ChIP-seq	Hsmm	H3K4me3	0.68	-0.29
424	Histone Modifications by ChIP-seq	Hsmm	H3K79me2	0.29	0.32
425	Histone Modifications by ChIP-seq	Hsmm	H3K9ac	0.32	-0.34
426	Histone Modifications by ChIP-seq	Hsmm	H3K9me3	0.28	0.35
427	Histone Modifications by ChIP-seq	Hsmm	H4K20me1	0.51	0.45
428	Histone Modifications by ChIP-seq	Hsmmtube	CTCF	0.46	0.64
429	Histone Modifications by ChIP-seq	Hsmmtube	EZH2_(39875)	0.49	-0.37

430	Histone Modifications by ChIP-seq	Hsmmtube	H2A.Z	0.59	0.32
431	Histone Modifications by ChIP-seq	Hsmmtube	H3K27ac	0.31	0.32
432	Histone Modifications by ChIP-seq	Hsmmtube	H3K27me3	1.08	-0.34
433	Histone Modifications by ChIP-seq	Hsmmtube	H3K36me3	-1.28	0.33
434	Histone Modifications by ChIP-seq	Hsmmtube	H3K4me1	-0.28	0.35
435	Histone Modifications by ChIP-seq	Hsmmtube	H3K4me2	0.53	0.29
436	Histone Modifications by ChIP-seq	Hsmmtube	H3K4me3	0.53	-0.30
437	Histone Modifications by ChIP-seq	Hsmmtube	H3K79me2	0.42	0.38
438	Histone Modifications by ChIP-seq	Hsmmtube	H3K9ac	0.31	-0.35
439	Histone Modifications by ChIP-seq	Hsmmtube	H3K9me3	1.03	0.79
440	Histone Modifications by ChIP-seq	Hsmmtube	H4K20me1	0.46	0.86
441	Histone Modifications by ChIP-seq	Huvec	CTCF	0.49	-0.32
442	Histone Modifications by ChIP-seq	Huvec	EZH2_(39875)	1.32	-0.45
443	Histone Modifications by ChIP-seq	Huvec	H2A.Z	0.56	0.37
444	Histone Modifications by ChIP-seq	Huvec	H3K27ac	0.40	-0.28
445	Histone Modifications by ChIP-seq	Huvec	H3K27me3	1.43	0.30
446	Histone Modifications by ChIP-seq	Huvec	H3K36me3	-0.86	-0.38
447	Histone Modifications by ChIP-seq	Huvec	H3K4me1	0.56	-0.33
448	Histone Modifications by ChIP-seq	Huvec	H3K4me2	0.55	-0.39
449	Histone Modifications by ChIP-seq	Huvec	H3K4me3	0.67	-0.45
450	Histone Modifications by ChIP-seq	Huvec	H3K79me2	-0.31	0.43
451	Histone Modifications by ChIP-seq	Huvec	H3K9ac	0.48	-0.35
452	Histone Modifications by ChIP-seq	Huvec	H3K9me1	0.58	1.12
453	Histone Modifications by ChIP-seq	Huvec	H3K9me3	-0.40	0.33
454	Histone Modifications by ChIP-seq	Huvec	H4K20me1	0.40	1.19
455	Histone Modifications by ChIP-seq	K562	CBP_(sc-369)	-0.43	-0.66
456	Histone Modifications by ChIP-seq	K562	CBX2	0.35	0.29
457	Histone Modifications by ChIP-seq	K562	CBX3_(SC-101004)	-1.22	-0.35
458	Histone Modifications by ChIP-seq	K562	CBX8	0.34	-0.45
459	Histone Modifications by ChIP-seq	K562	CHD1_(A301-218A)	-0.65	-0.29
460	Histone Modifications by ChIP-seq	K562	CHD4_Mi2	-1.50	0.31
461	Histone Modifications by ChIP-seq	K562	CHD7_(A301-223A-1)	-1.04	0.34
462	Histone Modifications by ChIP-seq	K562	CTCF	-0.43	0.34
463	Histone Modifications by ChIP-seq	K562	EZH2_(39875)	-0.44	0.33
464	Histone Modifications by ChIP-seq	K562	H2A.Z	-0.91	-0.32
465	Histone Modifications by ChIP-seq	K562	H3K27ac	-1.22	-0.35
466	Histone Modifications by ChIP-seq	K562	H3K27me3	0.71	0.64
467	Histone Modifications by ChIP-seq	K562	H3K36me3	-0.72	0.39
468	Histone Modifications by ChIP-seq	K562	H3K4me1	-1.25	0.30
469	Histone Modifications by ChIP-seq	K562	H3K4me2	-0.64	-0.30
470	Histone Modifications by ChIP-seq	K562	H3K4me3	-0.45	-0.35
471	Histone Modifications by ChIP-seq	K562	H3K79me2	-0.96	0.42
472	Histone Modifications by ChIP-seq	K562	H3K9ac	-0.91	-0.36
473	Histone Modifications by ChIP-seq	K562	H3K9me1	-0.63	0.31
474	Histone Modifications by ChIP-seq	K562	H3K9me3	0.74	0.60
475	Histone Modifications by ChIP-seq	K562	H4K20me1	-0.95	0.31
476	Histone Modifications by ChIP-seq	K562	HDAC1_(SC-6298)	-0.66	0.39
477	Histone Modifications by ChIP-seq	K562	HDAC2_(A300-705A)	-0.57	-0.59

478	Histone Modifications by ChIP-seq	K562	HDAC6_(A301-341A)	-0.62	-0.38
479	Histone Modifications by ChIP-seq	K562	LSD1	-0.72	-0.52
480	Histone Modifications by ChIP-seq	K562	NCoR	-0.42	0.33
481	Histone Modifications by ChIP-seq	K562	NSD2_(ab75359)	-1.71	0.33
482	Histone Modifications by ChIP-seq	K562	p300	-0.72	0.36
483	Histone Modifications by ChIP-seq	K562	PCAF	-0.50	-0.39
484	Histone Modifications by ChIP-seq	K562	PHF8_(A301-772A)	-0.47	-0.49
485	Histone Modifications by ChIP-seq	K562	PLU1	-0.91	-0.34
486	Histone Modifications by ChIP-seq	K562	RBBP5_(A300-109A)	-0.76	-0.43
487	Histone Modifications by ChIP-seq	K562	REST	0.32	0.36
488	Histone Modifications by ChIP-seq	K562	RNF2	-0.67	-0.63
489	Histone Modifications by ChIP-seq	K562	SAP30_(39731)	-0.44	-0.40
490	Histone Modifications by ChIP-seq	K562	SETDB1	-0.65	-0.37
491	Histone Modifications by ChIP-seq	K562	SIRT6	-1.85	0.34
492	Histone Modifications by ChIP-seq	K562	SUZ12_(05-1317)	-0.66	-0.45
493	Histone Modifications by ChIP-seq	Monocytes-CD14+	CTCF	-0.57	-0.56
494	Histone Modifications by ChIP-seq	Monocytes-CD14+	H2A.Z	0.38	0.33
495	Histone Modifications by ChIP-seq	Monocytes-CD14+	H3K27ac	-0.95	-0.30
496	Histone Modifications by ChIP-seq	Monocytes-CD14+	H3K27me3	1.87	0.35
497	Histone Modifications by ChIP-seq	Monocytes-CD14+	H3K36me3	-1.55	0.38
498	Histone Modifications by ChIP-seq	Monocytes-CD14+	H3K4me1	-0.28	0.28
499	Histone Modifications by ChIP-seq	Monocytes-CD14+	H3K4me2	0.54	0.51
500	Histone Modifications by ChIP-seq	Monocytes-CD14+	H3K4me3	0.33	-0.33
501	Histone Modifications by ChIP-seq	Monocytes-CD14+	H3K79me2	-0.46	-0.55
502	Histone Modifications by ChIP-seq	Monocytes-CD14+	H3K9ac	-0.49	-0.53
503	Histone Modifications by ChIP-seq	Monocytes-CD14+	H3K9me3	1.24	0.60
504	Histone Modifications by ChIP-seq	Monocytes-CD14+	H4K20me1	-0.42	-0.33
505	Histone Modifications by ChIP-seq	NH-A	CTCF	0.53	0.60
506	Histone Modifications by ChIP-seq	NH-A	EZH2_(39875)	0.91	0.63
507	Histone Modifications by ChIP-seq	NH-A	H2A.Z	0.77	0.39
508	Histone Modifications by ChIP-seq	NH-A	H3K27ac	0.48	0.39
509	Histone Modifications by ChIP-seq	NH-A	H3K27me3	1.64	0.28
510	Histone Modifications by ChIP-seq	NH-A	H3K36me3	-0.92	0.32
511	Histone Modifications by ChIP-seq	NH-A	H3K4me1	0.61	0.37
512	Histone Modifications by ChIP-seq	NH-A	H3K4me2	0.75	0.36
513	Histone Modifications by ChIP-seq	NH-A	H3K4me3	0.65	0.38
514	Histone Modifications by ChIP-seq	NH-A	H3K79me2	0.32	0.72
515	Histone Modifications by ChIP-seq	NH-A	H3K9ac	0.46	0.45
516	Histone Modifications by ChIP-seq	NH-A	H3K9me3	0.36	0.37
517	Histone Modifications by ChIP-seq	NH-A	H4K20me1	-0.34	0.35
518	Histone Modifications by ChIP-seq	Nhdf-Ad	CTCF	0.45	0.43
519	Histone Modifications by ChIP-seq	Nhdf-Ad	EZH2_(39875)	1.70	-0.58
520	Histone Modifications by ChIP-seq	Nhdf-Ad	H2A.Z	0.61	-0.29
521	Histone Modifications by ChIP-seq	Nhdf-Ad	H3K27ac	-0.42	0.31
522	Histone Modifications by ChIP-seq	Nhdf-Ad	H3K27me3	1.26	-0.67
523	Histone Modifications by ChIP-seq	Nhdf-Ad	H3K36me3	-0.56	0.31
524	Histone Modifications by ChIP-seq	Nhdf-Ad	H3K4me1	0.49	0.37
525	Histone Modifications by ChIP-seq	Nhdf-Ad	H3K4me2	0.63	-0.37

526	Histone Modifications by ChIP-seq	Nhdf-Ad	H3K4me3	0.59	-0.35
527	Histone Modifications by ChIP-seq	Nhdf-Ad	H3K79me2	-0.29	0.58
528	Histone Modifications by ChIP-seq	Nhdf-Ad	H3K9ac	-0.30	-0.32
529	Histone Modifications by ChIP-seq	Nhdf-Ad	H3K9me3	-0.44	0.83
530	Histone Modifications by ChIP-seq	Nhdf-Ad	H4K20me1	-0.96	0.31
531	Histone Modifications by ChIP-seq	Nhek	CTCF	-0.42	0.49
532	Histone Modifications by ChIP-seq	Nhek	EZH2_(39875)	0.77	0.34
533	Histone Modifications by ChIP-seq	Nhek	H2A.Z	0.44	-0.38
534	Histone Modifications by ChIP-seq	Nhek	H3K27ac	-0.46	-0.32
535	Histone Modifications by ChIP-seq	Nhek	H3K27me3	1.02	-0.50
536	Histone Modifications by ChIP-seq	Nhek	H3K36me3	-1.53	-0.39
537	Histone Modifications by ChIP-seq	Nhek	H3K4me1	-0.33	-0.30
538	Histone Modifications by ChIP-seq	Nhek	H3K4me2	0.41	0.31
539	Histone Modifications by ChIP-seq	Nhek	H3K4me3	0.59	-0.35
540	Histone Modifications by ChIP-seq	Nhek	H3K79me2	-0.36	0.46
541	Histone Modifications by ChIP-seq	Nhek	H3K9ac	-0.43	-0.28
542	Histone Modifications by ChIP-seq	Nhek	H3K9me1	-0.43	0.44
543	Histone Modifications by ChIP-seq	Nhek	H3K9me3	-0.60	0.66
544	Histone Modifications by ChIP-seq	Nhek	H4K20me1	-0.46	-0.39
545	Histone Modifications by ChIP-seq	Nhlf	CTCF	0.32	-0.36
546	Histone Modifications by ChIP-seq	Nhlf	EZH2_(39875)	1.43	-0.36
547	Histone Modifications by ChIP-seq	Nhlf	H2A.Z	0.46	-0.30
548	Histone Modifications by ChIP-seq	Nhlf	H3K27ac	-0.32	0.28
549	Histone Modifications by ChIP-seq	Nhlf	H3K27me3	0.78	0.29
550	Histone Modifications by ChIP-seq	Nhlf	H3K36me3	-1.32	0.34
551	Histone Modifications by ChIP-seq	Nhlf	H3K4me1	0.40	-0.42
552	Histone Modifications by ChIP-seq	Nhlf	H3K4me2	0.57	-0.43
553	Histone Modifications by ChIP-seq	Nhlf	H3K4me3	0.55	-0.33
554	Histone Modifications by ChIP-seq	Nhlf	H3K79me2	0.28	0.31
555	Histone Modifications by ChIP-seq	Nhlf	H3K9ac	0.33	-0.42
556	Histone Modifications by ChIP-seq	Nhlf	H3K9me3	-0.31	0.41
557	Histone Modifications by ChIP-seq	Nhlf	H4K20me1	0.31	0.72
558	Histone Modifications by ChIP-seq	Osteobl	CTCF	-0.34	0.46
559	Histone Modifications by ChIP-seq	Osteobl	H2A.Z	0.64	0.39
560	Histone Modifications by ChIP-seq	Osteobl	H3K27ac	0.40	0.36
561	Histone Modifications by ChIP-seq	Osteobl	H3K27me3	1.10	-0.44
562	Histone Modifications by ChIP-seq	Osteobl	H3K36me3	-0.87	0.60
563	Histone Modifications by ChIP-seq	Osteobl	H3K4me1	0.72	0.40
564	Histone Modifications by ChIP-seq	Osteobl	H3K4me2	0.82	0.29
565	Histone Modifications by ChIP-seq	Osteobl	H3K4me3	0.76	-0.34
566	Histone Modifications by ChIP-seq	Osteobl	H3K79me2	-0.31	0.72
567	Histone Modifications by ChIP-seq	Osteobl	H3K9me3	0.28	0.40
568	Histone Modifications by ChIP-seq	Osteobl	H4K20me1	-0.42	0.49
569	Histone Modifications by ChIP-seq	Osteobl	P300_KAT3B	1.37	0.60

Note: Epigenomic elements enriched with *P* -value < 0.01 are highlighted in red while depleted epigenomic elements with *P* -value < 0.01 are highlighted in green.

Table S4. Cell types used to obtain cell type-specific epigenomic elements.

Cell	Description	Lineage	Tissue	Karyotype	Sex	Vendor ID	*Label*
A549	epithelial cell line derived from a lung carcinoma tissue. (PMID: 175022), "This line was initiated in 1972 by D.J. Giard, et al. through explant culture of lung carcinomatous tissue from a 58-year-old caucasian male." - ATCC, newly promoted to tier 2: not in 2011 analysis	endoderm	epithelium	cancer	M	ATCC CCL-185	A549
CD20+	B cells from donors RO01778 and RO01794, newly promoted to tier 2: not in 2011 analysis	mesoderm	blood	normal	F	Dr. Heimfeld Lab at FHCRC CD20+	*B-cells CD20+*
CD20+_RO01794	B cells, African American, draw number 1, newly promoted to tier 2: not in 2011 analysis	mesoderm	blood	normal	F	Rajinder Kaul, UW CD20+_RO01794	*B cells CD20+ RO01794*
Dnd41	T cell leukemia with Notch mutation	mesoderm	blood	cancer	M	DSMZ Dnd41	Dnd41
GM12878	B-lymphocyte, lymphoblastoid, International HapMap Project - CEPH/Utah - European Caucasion, Epstein-Barr Virus	mesoderm	blood	normal	F	Coriell GM12878	GM12878
H1-hESC	embryonic stem cells	inner cell mass	embryonic stem cell	normal	M	WiCell Research Institute WA01	H1-hESC
HeLa-S3	cervical carcinoma	ectoderm	cervix	cancer	F	ATCC CCL-2.2	HeLa-S3
HepG2	hepatocellular carcinoma	endoderm	liver	cancer	M	ATCC HB-8065	HepG2
HMEC	mammary epithelial cells	ectoderm	breast	normal	U	Lonza CC-2551	HMEC
HSMM	skeletal muscle myoblasts	mesoderm	muscle	normal	U	Refer to the protocol documents for differing sources CC-2580	HSMM
HSMMtube	skeletal muscle myotubes differentiated from the HSMM cell line	mesoderm	muscle	normal	U	Lonza See HSMM	HSMMtube
HUVEC	umbilical vein endothelial cells	mesoderm	blood vessel	normal	U	Lonza CC-2517	HUVEC
K562	leukemia, "The continuous cell line K-562 was established by Lozzio and Lozzio from the pleural effusion of a 53-year-old female with chronic myelogenous leukemia in terminal blast crises." - ATCC	mesoderm	blood	cancer	F	ATCC CCL-243	K562
NH-A	astrocytes (also called Astrocy)	ectoderm	brain	normal	U	Lonza CC-2565	NH-A
NHDF-Ad	adult dermal fibroblasts	mesoderm	skin	normal	F	Lonza CC-2511, CC-2511T225	NHDF-Ad
NHEK	epidermal keratinocytes	ectoderm	skin	normal	U	Lonza CC-2501	NHEK

NHLF	lung fibroblasts	endoderm	lung	normal	U	Lonza CC-2512	NHLF
Osteobl	osteoblasts (NHOst)	mesoderm	bone	normal	U	Lonza CC-2538	*Osteoblasts*
Monocytes-CD14+ _RO01746	Monocytes-CD14+ are CD14-positive cells from human leukapheresis production, from donor RO 01746 (draw 1 ID is RO 01746, draw 2 ID is RO 01826), Monocytes-CD14+_RO01746 and Monocytes-CD14+_RO01826 are being used as replicates, newly promoted to tier 2: not in 2011 analysis	mesoderm	monocytes	normal	F	S. Heimfeld Laboratory, FHCRC Monocytes-CD14+_RO01746	*Monocytes CD14+ RO01746*

Table S5. siRNA sequences for *BDNF*.

siRNA		Sequence
siRNA1	Sense	CAGUAUUUCUACGAGACCATT
	Antisense	UGGUCUCGUAGAAAUACUGTT
siRNA2	Sense	CGAUUCAUAAGGAUAGACATT
	Antisense	UGUCUAUCCUUAUGAAUCGTT

Table S6. Primer sequences for PCR analyses.

Gene		Primer sequence
BDNF	Forward	GATGCCGCAAACATGTCTATGA
	Reverse	TAATACTGTCACACACGCTCAGCTC
ALP	Forward	ACACCTTGACTGTGGTTACTGCTGA
	Reverse	CCTTGTAGCCAGGCCCGTTA
COL1	Forward	GGGGCAAGACAGTCATCGAA
	Reverse	GGTGGGAGGGAACCAGATTG
RUNX2	Forward	CGCCCCTCCCTGAACTCT
	Reverse	TGCCTGCCTGGGATCTGTA
OCN	Forward	CTGACAAAGCCTTCATGTCCAA
	Reverse	GCGCCGGAGTCTGTTCACTA
GAPDH	Forward	ACTGAGGACCAGGTTGTC
	Reverse	TGCTGTAGCCGTATTCATTG
